# Genome sequence reveals that *Pseudomonas fluorescens* F113 possesses a large and diverse array of systems for rhizosphere function and host interaction

**DOI:** 10.1186/1471-2164-14-54

**Published:** 2013-01-25

**Authors:** Miguel Redondo-Nieto, Matthieu Barret, John Morrissey, Kieran Germaine, Francisco Martínez-Granero, Emma Barahona, Ana Navazo, María Sánchez-Contreras, Jennifer A Moynihan, Candela Muriel, David Dowling, Fergal O’Gara, Marta Martín, Rafael Rivilla

**Affiliations:** 1 Departamento de Biología, Facultad de Ciencias, Universidad Autónoma de Madrid, c/Darwin, 2, Madrid, 28049, Spain; 2 BIOMERIT Research Centre, Microbiology Department, University College Cork, Cork, Ireland; 3 Microbiology Department, Food Science Building, University College Cork, Cork, Ireland; 4 Institute of Technology, Carlow, Ireland

## Abstract

**Background:**

*Pseudomonas fluorescens* F113 is a plant growth-promoting rhizobacterium (PGPR) isolated from the sugar-beet rhizosphere. This bacterium has been extensively studied as a model strain for genetic regulation of secondary metabolite production in *P. fluorescens*, as a candidate biocontrol agent against phytopathogens, and as a heterologous host for expression of genes with biotechnological application. The F113 genome sequence and annotation has been recently reported.

**Results:**

Comparative analysis of 50 genome sequences of strains belonging to the *P. fluorescens* group has revealed the existence of five distinct subgroups. F113 belongs to subgroup I, which is mostly composed of strains classified as *P. brassicacearum*. The core genome of these five strains is highly conserved and represents approximately 76% of the protein-coding genes in any given genome. Despite this strong conservation, F113 also contains a large number of unique protein-coding genes that encode traits potentially involved in the rhizocompetence of this strain. These features include protein coding genes required for denitrification, diterpenoids catabolism, motility and chemotaxis, protein secretion and production of antimicrobial compounds and insect toxins.

**Conclusions:**

The genome of *P. fluorescens* F113 is composed of numerous protein-coding genes, not usually found together in previously sequenced genomes, which are potentially decisive during the colonisation of the rhizosphere and/or interaction with other soil organisms. This includes genes encoding proteins involved in the production of a second flagellar apparatus, the use of abietic acid as a growth substrate, the complete denitrification pathway, the possible production of a macrolide antibiotic and the assembly of multiple protein secretion systems.

## Background

*Pseudomonas fluorescens* F113 is a PGPR strain isolated from the sugar-beet rhizosphere in Ireland [1]. Initially this strain was selected and studied because of its capacity to inhibit growth of a range of phytopathogenic bacteria, fungi, oomycetes and nematodes including *Pectobacterium caratovorum*[2], *Fusarium oxysporum*[3]*, Pythium ultimum*[4] and *Globodera spp.*[5]. This antimicrobial capacity is strongly linked to the production of a secondary metabolite, 2,4-diacetylphloroglucinol (DAPG) [4,6], and renders the strain a candidate bio control microorganism for agrobiotech applications. The biosynthetic and regulatory genes required for the synthesis of this polyketide are located in an 8 kb cluster of nine protein-coding genes that are highly conserved in other DAPG-producing *P. fluorescens* but completely absent from non-producing strains [7,8]. In addition, *P. fluorescens* F113 is an excellent rhizosphere coloniser of different plant species including wheat [9], alfalfa [10], and willow [11,12]. For that reason, F113 is widely used as a model for studying rhizosphere colonization [13,14]. Several studies have sought to take advantage of this colonising ability by developing novel genetically modified derivatives with biotechnological traits, for example the capacity to degrade polychlorinated biphenyls and other environmental pollutants [11,15].

The *Pseudomonas* genus is composed of more than 100 species, which have been divided by multilocus sequence analysis (MLSA) into nine major groups: *P. fluorescens*, *P. syringae*, *P. lutea*, *P. putida*, *P. anguilliseptica*, *P. straminea*, *P. aeruginosa*, *P. oleovorans* and *P. stutzeri*[16]. Some of these groups are themselves composed of different subgroups containing multiple species. For instance, the *P. fluorescens* group can be further divided into nine subgroups [16]. The number of species present in each of these subgroups is somewhat difficult to assess since novel formal species, such as *P. protegens*[17], are frequently described. A number of genome sequences for strains belonging to the *P. fluorescens* group have been recently obtained (Additional file 1). The initial comparative genomic analysis of the three first complete genome sequences of strains belonging to the *P. fluorescens* group has highlighted a large number of strain-specific genes [18]. Therefore, it has been proposed that the sequenced strains belong to a complex of species rather than to a single species [19]. More recently, a comparative genomics analysis performed on 10 genome sequences of strains belonging to the *P. fluorescens* group has highlighted three main subclades [20].

Here we present an analysis of the genome sequence of *P. fluorescens* F113 [21] compared to 49 other complete and draft genomes of strains classified as *Pseudomonas* spp., *P. brassicacearum*, *P. fluorescens*, *P. protegens, P. mandelii*, *P. chlororaphis*, *P. tolaasii* and *P. extremaustralis*. The genome of *P. fluorescens* F113 [21] is composed of a single circular chromosome of 6,845,832 bp with an average G + C content of 60.8% and an overall coding density of 86.7% (Additional file 2). Following automatic annotation and subsequent manual curation, a total of 5862 protein-coding sequences, nine ncRNAs, five rRNA operons and 66 tRNA loci were detected in the genome of this strain. The F113 genome contains a variety of protein-coding genes that appear relevant for thriving in the rhizosphere environment. This includes unusual metabolic adaptations within the *P. fluorescens* species, protein-coding genes related to motility, genes encoding putative toxins targeted to diverse organisms and a very large number of protein-coding genes involved in the assembly of different secretion systems. The distribution of these traits in other strains belonging to the *P. fluorescens* group as well as the functionality of several of these genes are described in this manuscript.

## Results and discussion

### *P.fluorescens* F113 phylogenomics comparison

**Figure 1:**
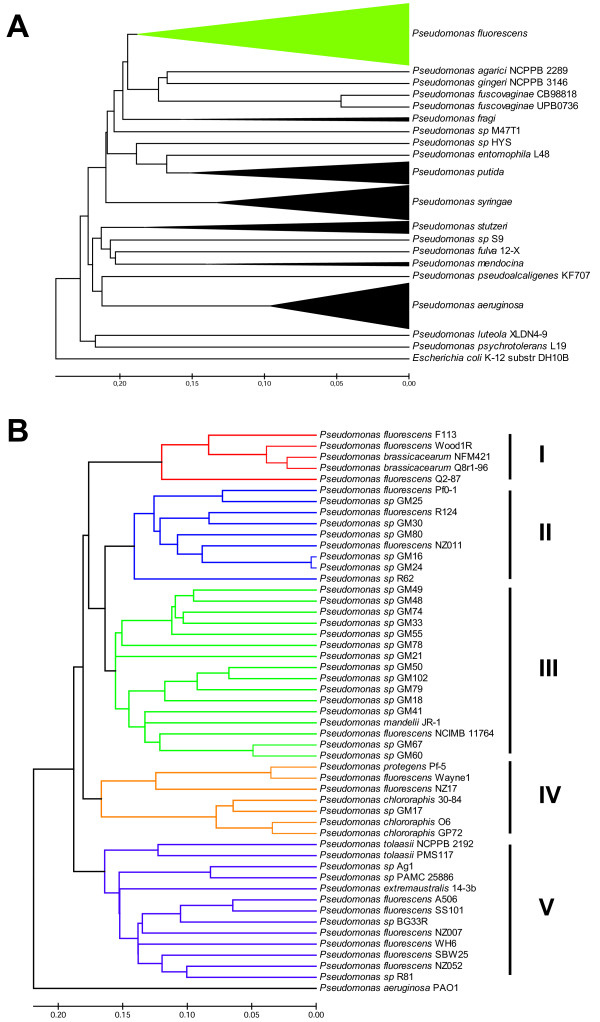
***Pseudomonas * spp phylogenomic analysis.** (**A**) Whole-genome based phylogenomic tree by using composition vector approach. Strains belonging to the * P. fluorescens * group are collapsed within the green branch. (**B**) Phylogenomic tree of strains in the * P. fluorescens * group. Subgroups I to V as described in this work, are highlighted in different colours. Scale shows kmer distribution divergence. Completed and draft genomes of the used * P. fluorescens * strains are listed in Additional file 1 .

In order to determine the phylogenetic relationship of F113 to other pseudomonads, a phylogenomic analysis of 162 *Pseudomonas* genomes and draft genomes available at the time of this writing was performed with the composition vector method [22]. The phylogenomics tree generated with such approach (Figure 1A) is mostly congruent with previous concatenated sequence trees obtained in different MLSA analyses [16,20,23]; and clearly highlighted the need to re-evaluate the taxonomic status of species belonging to the *P. fluorescens* group [18]. Compared to other trees, the major difference is the phylogenetic position of *P. putida* and *P. syringae* with respect to the *P. fluorescens* group. However the distances shown here for this two species to the parent node are very small and differences could be due to the different methods used. Fifty strains (Additional file 1) previously classified as *P. fluorescens* (including F113)*, P. brassicacearum*, *P. protegens, P. mandelii*, *P. chlororaphis*, *P. tolaasii*, *P. extremaustralis* and *Pseudomonas* spp. formed a single cluster which branched significantly deeper than other clusters that represent defined species such as *P. aeruginosa* or *P. syringae.* Detailed phylogenomic analysis of the *P. fluorescens* group revealed the presence of at least five subgroups with strains previously classified as *P. fluorescens*, interspersed with strains classified in other species (Figure 1B). The analysis of the whole 50 strains ORFome, represented as a vector of 6mer peptide frecuencies, has allowed us to increase the number of *P. fluorescens* subgroups from three [20] to five. It is likely that some of these subclusters may represent different species, especially considering that all of these subclusters branch above a 0.16 dissimilarity threshold, a value enough to discriminate recognized species within the genus *Pseudomonas*.

Subgroup I contains F113 together with strains classified as *Pseudomonas brassicacearum*, including the complete genome sequence of NFM421 [24]. Subgroup II contains the sequenced strain *P. fluorescens* Pf0-1 and other strains classified either as *P. fluorescens* or *Pseudomonas* spp*.* Although, these two subgroups were initially described as one subclade by Loper et al. [20], the separation between these two subgroups is supported by a branching above 0.16 dissimilarity. Subgroup III includes *P. fluorescens* NCIMB 11764, *P. mandelii* JR-1 and several unclassified pseudomonads, which were not analysed in previous studies. Subgroup IV contains *P. protegens* Pf-5, all the *P. chlororaphis* strains, *P. fluorescens* strains Wayne1 and NZ17 and one unclassified pseudomonad. This subgroup is congruent with subcluster 1 [20]. Finally, subgroup V contains *P. fluorescens* strains SBW25, WH6, A506, SS101 NZ007 and NZ052, together with *P. tolaasii* strains, *P. extremaustralis* and four unclassified pseudomonads. This subgroup corresponds to subcluster 3 [20].

**Figure 2:**
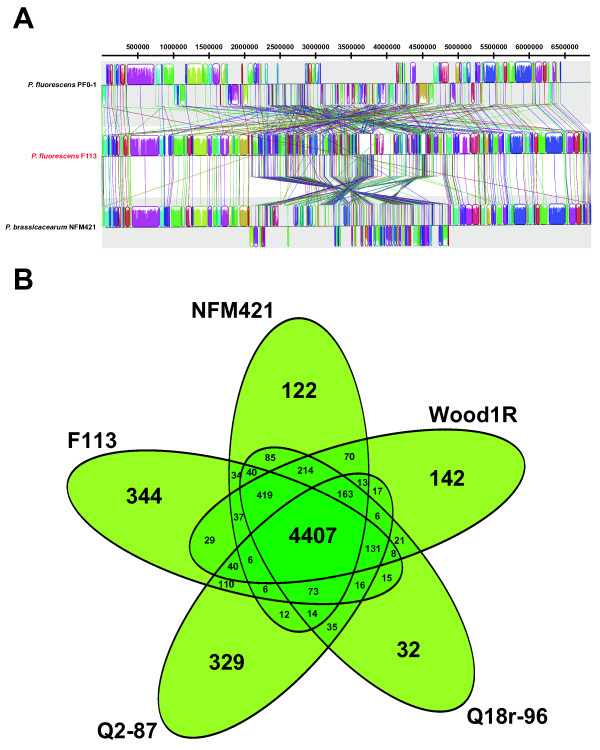
**Comparison of *P. fluorescens * F113 genome and CDSs against other pseudomonads sequences and ORFomes.** (**A**) Genome to genome alignment of * P. fluorescens * F113, * P. fluorescens * Pf0-1 and * P. brassicacearum * NFM421, using Mauve software with a window of 1000 nucleotides and F113 as the reference genome. Boxes with same colour indicate syntenic regions. Boxes below the horizontal strain line indicate inverted regions. Rearrangements are shown by coloured lines. Scale is in nucleotides. (**B**) Venn diagram showing the number of clusters of orthologous CDSs shared and unique between strains clustered in Subgroup I: * P. fluorescens * F113, WoodR1, Q2-87, and * P. brassicacearum * NFM421 and Q8r1-96.

The genome sequence of strain F113 was then aligned with its closest fully sequenced relatives, *P. brassiccacearum* NFM421 and *P. fluorescens* Pf0-1 (Figure 2A). This alignment shows that F113 genome is much more similar to the NFM421 than to the Pf0-1 genome, supporting the subclustering described above. It also shows that the major difference between the F113 and NFM421 genomes is a large inversion in the distal part of the genome, opposite to the replication origin. It is interesting to note that both strains were isolated from a similar ecosystem, the rhizosphere of a crop although in different hemispheres and that both strains share similarities such as the occurrence of phase variation during rhizosphere colonization, the presence of denitrification genes (see below) and the presence of three chemotaxis gene clusters (see below).

The presence of the F113 orthologous coding sequences (CDSs) in the 50 genomes sequences from the *P. fluorescens* group was assessed using OrthoMCL [25]. Following this analysis, the core genome of the *P. fluorescens* group is composed of 2003 orthologous CDSs present in all the fifty strains examined. Unsurprisingly, the size of the core genome is smaller in comparison to previous analysis performed with 3 (3642 orthologous CDSs) and 10 (2789 orthologous CDSs) genome sequences [18,20]. The core genome of the *P. fluorescens* subgroup I is composed of 4407 orthologous CDS (Figure 2B), which represents approximately 76% of the whole predicted proteome. In addition 344 orthologous CDSs (5.9% of the predicted proteome) found in the F113 genome are not present in any other genome of strains within this subgroup. These 344 CDSs were subjected to BLASTP analysis against the non-redundant (nr) NCBI database (Additional file 3). Closest relatives of these unique protein-coding genes are mostly found in other gamma-proteobacterial genomes, including other *Pseudomonas* species, and could be involved in important functions relevant for rhizosphere fitness (discussed in the sections below).

### The F113 genome encodes important traits involved in rhizocompetence

**Figure 3:**
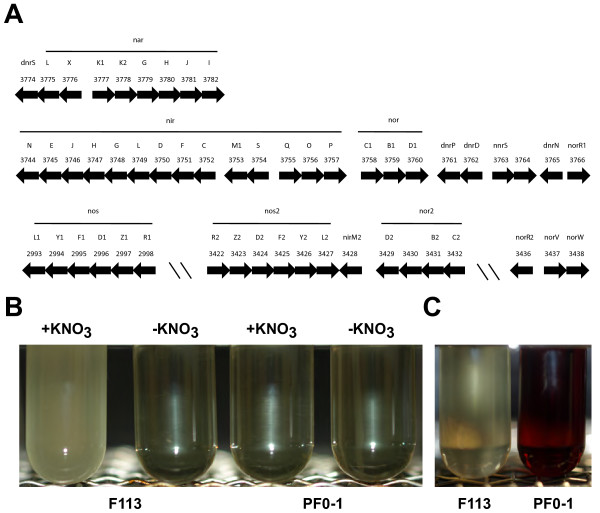
**Denitrification in *P. fluorescens * F113.** (**A**) Denitrification genes organization in * P. fluorescens * F113. Numbers above arrows indicate feature code (PSF113_XXXX). (**B**) Growth test in anaerobic conditions of * P. fluorescens * F113 and Pf0-1 in the presence or absence of KNO_3_ as electron acceptor. Growth is observed as turbidity. (**C**) NaNO_2_ depletion measured by NitriVer® 5 reagent. Red colour shows the presence of nitrite in the medium.

A number of plant-associated bacteria, including *Pseudomonas* spp. [26,27] and *Azospirillum brasilense*[28], are able to use nitrogen oxides as alternative electrons acceptors under oxygen limiting conditions, a process called denitrification. Denitrification has been shown to play a role in rhizosphere colonization, since mutants of *P. fluorescens* impaired in nitrate or nitrite reductases are deficient in the colonization of the rhizosphere [26,29,30]. Furthermore, it has been shown that denitrification is statistically associated with rhizosphere competence in rhizosphere isolated fluorescent pseudomonads [31]. The F113 genome encodes approximately 50 proteins involved in denitrification. These genes are organized in four clusters on the chromosome (Figure 3A). The first cluster (PSF113_3777-3782) contains the *narK1K2GHJI* genes, which encode the membrane-bound nitrate reductase. The sensor-response regulator NarL-NarX and the transcriptional regulator DnrS are encoded upstream of the *nar* genes. The periplasmic (Nap) and the assimilatory nitrate reductase (Nas) are also present in the genome of F113 (PSF113_3040-3044 and PSF113_1771, respectively). The second cluster (PSF113_3744-3766) is composed of the *nir* and *nor1* genes encoding nitrite and nitric oxide reductase, respectively. The third cluster (PSF113_2993-2998) contains the *nos1* genes that encode the nitrous oxide reductase. All these genes showed highest homology with denitrification genes in different pseudomonads (Additional file 4). Finally the last cluster (PSF113_3422-PSF113_3432) contains the *nos2* and *nor2* genes. Although the *nos2* genes showed homology (73-85% identity) with the *nos1* genes, the *nor2* genes are quite divergent to *nor1* genes and showed higher homology with genes encoded in *Acidovorax* and several *Enterobacteriaceae*. It is interesting to note that the σ54-transcriptional activator NorR2 (PSF113_3436) is encoded in the downstream region of the *nos2nor2* cluster, which suggests that *nos1nor1* and *nos2nor2* genes might be independently regulated. In order to test the functionality of these denitrification genes, the growth of F113 under anaerobic conditions was compared to the growth of Pf0-1, which does not contain denitrification genes. While Pf0-1 did not grow under these conditions, F113 was able to grow anaerobically using nitrate and nitrite as electron acceptors (Figure 3B). Furthermore, a total depletion of nitrite was observed after 48 h of growth (Figure 3C). These results together with the presence of two copies of the *nos* genes, strongly indicate that F113 is capable of full denitrification. The duplication of genes encoding nitric oxide reductase and nitrous oxide reductase might be related to high denitrification rates, as has been shown for a *Bacillus* strain that contains two different copies of the *nosZ* gene and showed high amounts of N_2_ production [32]. The presence of denitrification genes was analyzed in the fifty strains forming the *P. fluorescens* group (Additional file 4). While all strains of subgroup I contained a complete denitrification pathway, none of the strains of subgroup II possessed denitrification genes, which further support the distinction between subgroup I and II. Interestingly some denitrification genes are present in few strains belonging to subgroups III, IV and V, which might indicate horizontal gene transfer. Whereas *nor2* genes were only present F113 and NFM421, *nos2* genes were systematically found in strains harbouring the *nos1* genes.

Another metabolic adaption is the potential degradation of aromatic diterpenes encoded in the F113 genome. Aromatic diterpenes are tricyclic resin acids naturally produced by trees and include abietic, dehydroabietic and palustric acids. Bacteria that harbor the *dit* genes can grow in these substrates as the sole carbon and energy source [33]. These genes have been found in several *Proteobacteria* such as *Burkholderia*, *Cupriavidus* and *Pseudomonas*[33]. Among *Pseudomonas*, *dit* genes have been found in *P. abietaniphila*[34], *P. diterpeniphila*[35] and in *P. aeruginosa* strain PA2192 [36]. F113 chromosome harbors a 65 Kb genetic island containing the *dit* genes (PSF113_3386-PSF113_3413). This region is contiguous to the *nos2-nor2* cluster and has a gene organization similar to the genetic island found in *P. aeruginosa* PA2192, although in this strain certain *dit* genes are duplicated and the genomic island is 115 Kb long [36]. Growth of F113 on abietic acid was compared with growth of Pf0-1. After twenty days, growth was observed for F113 but not for Pf0-1, suggesting that *dit* genes in F113 are functional.

### Plant growth-promoting traits

Previous studies have shown that *P. fluorescens* F113 is capable of inorganic phosphate solubilisation through the extracellular production of gluconic acid [37,38]. Moreover, F113 seems capable of mineralizing insoluble organic phosphates pool by the production of specific enzymes, such as the homologues of the alkaline phosphatases PhoD (PSF113_0888) and PhoX (PSF113_5402) of *P. fluorescens* Pf0-1 [39] and a beta-propeller phytase (PSF113_2886) [40]. Altogether, these traits could contribute to mobilization of insoluble soil phosphate into bioavailable forms that can be taken up by the plant root.

ACC deaminase activity in F113 has been reported previously [41] and analysis of the genome sequence indicates that PSF113_3500 (*acd*S) encodes a 1-aminocyclopropane-carboxylate (ACC) deaminase. ACC deaminase (EC 4.1.99.4) catalyses the degradation of the ethylene precursor, ACC, into ammonium and α-ketobutyrate (2-oxobutanoic acid) and has been linked to plant growth promotion activity in rhizosphere microorganisms [42]. The activity of the AcdS enzyme in F113 is 4.783 μM mg^-1^ protein hr^-1^, almost twice the activity of *Enterobacter* UW4 [43]. The expression of *acdS* is probably regulated by the LRP-like transcriptional regulator *acdR*, which is located upstream of *acdS*.

### Motility and chemotaxis

**Figure 4:**
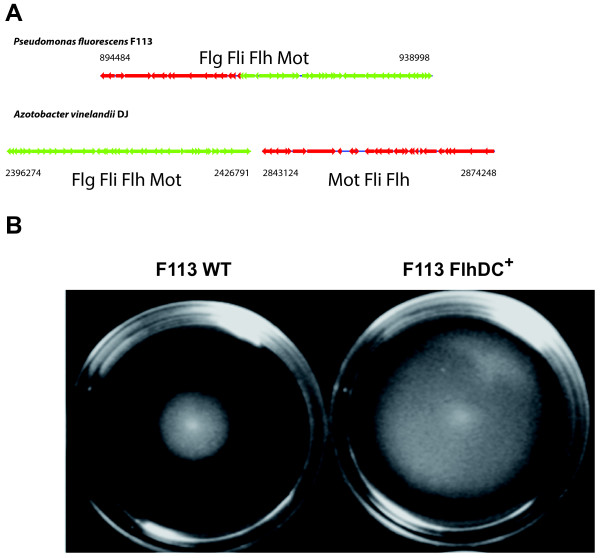
**F113 produces a second flagellar apparatus.** (**A**) Gene cluster organization of *P. fluorescens* F113 second flagellum compared to *Azotobacter vinelandii* DJ. Flagellar genes in *A. vinelandii* are located in two separated clusters compared to the single cluster in F113. ORFs arrows are at scale. Numbers indicate chromosome coordinates in both strains. (**B**) Swimming motility of *P. fluorescens* F113 wild type and a modified strain overexpressing the second flagellum master regulator *flhDC* operon. Increased motility is shown by a larger halo after 18 h. incubation.

Like all *Pseudomonas* strains, F113 genome encodes genes for the synthesis of polar flagella distributed in three clusters. Several of these genes in F113 have been previously analyzed [44,45] and regulation of flagella synthesis requires the master regulatory gene *fleQ*[46]. Besides these genes, F113 chromosome harbors another 41 kb flagella locus containing 40 CDSs (PSF113_0738-PSF113_0782), which is not present in any other *Pseudomonas* genome, with the exception of *P. extremaustralis* (Figure 4A). Conversely to the operons encoding flagellar genes in pseudomonads, this region contains an *flhDC* operon, encoding master regulatory proteins of flagella synthesis in other gamma-proteobacteria such as enterobacteria and *Azotobacter.* The 40 ORFs showed high homology to flagellar genes of *Azotobacter vinelandii*, a soil bacterium phylogenetically related to the genus *Pseudomonas*, but producing a different type of flagella, which are peritrichous, instead of the typical single or double polar flagella produced by pseudomonads. The region also showed synteny with the flagellar genes of *A. vinelandii* (Figure 4A). The *A. vinelandii* chromosome harbors flagellar genes in two clusters, I and II. Genes in cluster I are conserved in the same order in the 41 Kb region in the F113 chromosome. Cluster II in *A. vinelandii* is located 416 Kb downstream of Cluster I. A reduced version of this region and lacking 12 ORFs is located in an inverted orientation adjacent to Cluster I in the F113 chromosome. All the genes present in *A. vinelandii* clusters, but absent in the F113 genome, encode chemotaxis proteins such as CheZ, CheY, and CheB or proteins that are not essential for flagella synthesis, such as RfbC, RfbG and RfbF. Ectopic expression of *flhDC* in F113 increased swimming motility (Figure 4B) and resulted in hyperflagellation with multiple polar flagella (data not shown). These results indicate that F113 can produce the second type flagella, and that these flagella are functional. In *Rhodobacter sphaeroides*, a second acquired flagellum is also functional and coexists with the endogenous flagellar system [47]. In this case, as in F113, both flagella were polar. Although flagella whose synthesis is regulated by the master regulatory genes *flhDC* are generally peritrichous, regulation of polar flagella by this system has also been observed in *Burkholderia glumae*[48], indicating that *flhDC* regulation does not imply peritrichous flagellation.

**Figure 5:**
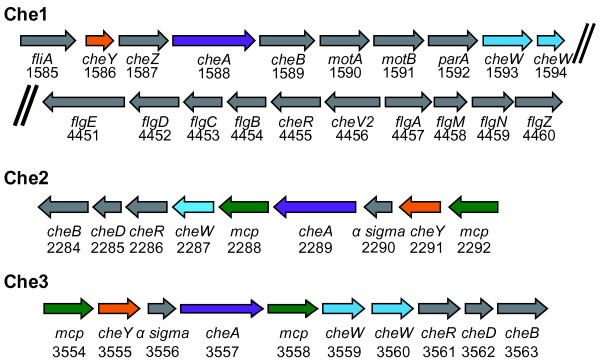
**The F113 genome contains three groups of *che * genes.** Genomic organisation of the Che regions in *P. fluorescens* F113. The Che1 genes are organized in two genomic regions. Arrows with the same colour correspond to paralogues. Numbers below arrows indicate feature code (PSF113_XXXX).

The F113 genome encodes five chemotaxis-like systems: Wsp, Chp, Che1, Che2 and Che3 (Additional file 5). The Wsp system (PSF113_1084-PSF113_1090) is implicated in regulation of motility and biofilm formation in F113 [13,49] and other *P. fluorescens* strains [50,51]. The Chp system (PSF113_5524-PSF113_5525) is located in the immediate vicinity of to *pil* genes and has been shown to control twitching motility in *Pseudomonas aeruginosa*[52]. Besides, the F113 genome encodes three Che systems (Figure 5). Che1 (PSF113_1586-PSF113_1594, PSF113_4455 and PSF113_4456) is located in the vicinity of protein-coding genes involved in the assembly of the endogenous flagellum. Mutation of the *cheA1* gene in F113 resulted in impaired motility and rhizosphere colonization [53] indicating that this system participates in chemotactic motility. It is interesting to note that no genes encoding methyl accepting chemotactic sensors (MCPs) are genetically linked to this system, although more than thirty ORFs predicted to encode MCP-like proteins are scattered in the F113 genome. The Che2 (PSF113_2284-PSF113_2292) and Che3 (PSF113_3554-PSF113_3563) systems are present in a restricted number of strains of the *P. fluorescens* group (Additional file 5). The Che 2 genes are only present in all strains of Subgroup I and three strains of the Subgroup V. The Che 3 genes are present only in three strains of Subgroup I: F113, NFM421 and Wood 1. Both Che 2 and Che 3 systems harbor two genes putatively encoding MCPs, suggesting that these systems might be implicated in specialized functions. This diversity of chemotaxis systems, as well as the production of a second type flagellum, highlights the importance of motility for F113. It is important to note that chemotaxis and motility mutants are among the most affected mutants in competitive rhizosphere colonization [44,53] and that the rhizospheric environment selects for hypermotile phenotypic variants [14,54] that are frequently hypercompetitive [14]. It also raises interesting questions about which chemotaxis systems are used by the second type flagellum and about motility under different physiological condition, for example anaerobic growth using nitrate or nitrite as electron acceptors.

### Protein and macromolecule secretion

Gram negative bacteria rely on several secretion systems to influence their environment by translocating protein and DNA into neighbouring cells and the extracellular milieu. These secretion systems can range from simple transporters to multi-component complexes and have been classified into six types: from type I through type VI secretion system (T1SS-T6SS) [55]. The genome of F113 contains a wide variety of secretion systems, which include six T1SSs, two T2SSs, two T3SSs, one T4SS, five T5aSSs, four T5bSS, one T5dSS and three T6SSss (Table 1).

**Table 1:** **The full repertoire of secretion systems of *P. fluorescens *F113**

**Secretion system**	**Phylogenetic cluster**	**Locus**	**Putative substrate**
T1SS		PSF113_0209-0211	PSF113_0208
		*opmH* (PSF113_0530)*	
		PSF113_1508-1510	PSF113_1511
		PSF113_2734-2736	PSF113_2737
		*aprDEF* (PSF113_2945-2947)	*aprA* (PSF113_2949), *aprI* (PSF113_2948), *lipA* (PSF113_2944)
		PSF113_3005-3007	*psmE* (PSF113_3004)
		PSF113_3055-3056*	
		PSF113_3303-3305	
T2SS	Xcp	*xcpP-Z* (PSF113_0437-0447), *xcpA* (PSF113_5006)	PSF113_0435, PSF113_0610, PSF113_2337, PSF113_3279
	Hxc	*hxcP-Z (*PSF113_3690-3700)	*psp* (PSF113_3701)
T3SS	SPI-I	*invF*-*orgB* (PSF113_1778-1801)	PSF113_1802
	Hrp1	*rspS*-*rspL* (PSF113_5585-5610)	*ropAA-1* (PSF113_1126), *ropAA-2* (PSF113_3486), *ropB* (PSF113_5598), *ropM* (PSF113_5616)
T4SS	GI-like	PSF113_3314-3334	
T5aSS		PSF113_2779	
		PSF113_2780	
		PSF113_4399	
		*estA* (PSF113_5339)	
		PSF113_5848	
T5bSS		PSF113_0792-0793	
		PSF113_1489-1490	
		PSF113_4466-4468	
		PSF113_2623-PSF113_3702	
T5dSS		*plpD* (PSF113_1517)	
T6SS	I	HSI-II (PSF113_5815-5833)	*hcp2* (PSF113_1976), PSF113_0495, PSF113_0666, PSF113_3144, PSF113_3904
	III	HSI-I (PSF113_5785-5808)	*vgrG1b* (PSF113_2885)
	IVA	HSI-III (PSF113_2407-2422)	

#### T2SSs

T2SS is the most ubiquitous secretion system used by Gram negative bacteria to secrete extracellular protein. Therefore, T2SS is well-conserved and involves a set of 11 to 12 proteins [56]. According to XcpR phylogenetic analysis, *Pseudomonas* T2SSs could be divided in two main clusters: Xcp (extracellular protein), and Hpx (homologue to Xcp) (Additional files 6 and 7). Two complete T2SSs related to the Xcp cluster (PSF113_0437-0447) and to the Hxc cluster (PSF113_3690-3700) are present in the genome of F113 (Additional file 7). The genetic organization of the Hxc cluster of F113 is highly similar with the Hxc cluster of *P. aeruginosa* PAO1 and *P. fluorescens* SBW25 [57,58]. Moreover, a predicted ORF sharing 74% and 72% identity at the amino acid level with the Hxc substrate Psp of *P. fluorescens* SBW25 [58] and DinG of *P. aeruginosa* MDR1 [59] is located immediately downstream of the Hxc locus, which suggest that this protein could possibly be secreted by the Hxc secretion system of F113. The second T2SS locus of F113 is identical to the second Xcp of *P. aeruginosa* PA7, which is present on the genomic island RGP69 (PSPA7_1407-1420) [60]. Based on homologies with known Xcp effectors, it seems that at least four predicted F113 proteins could potentially be Xcp substrates (PSF113_0435, PSF113_0610, PSF113_2337 and PSF113_3279).

#### T3SSs

T3SS is a nanomachine composed of approximately 25 proteins [61], which has evolved into seven different families: Ysc, Hrp1, Hrp2, SPI-1, SPI-2, *Rhizobiaceae* and *Chamydia*[62,63]. The distribution of T3SS loci in complete or draft *Pseudomonas* genome sequences was investigated using the following ORFs PA1703, PSPTO_1402, PSPPH_2520 and PSF113_1781 (COG4789) as queries in sequential BLASTp and TBLASTn searches. Four T3SS phylogenetic families, Ysc, SPI-1, Hrp1 and Rhizobia are encoded in the different *Pseudomonas* chromosomes (Additional file 8). While Ysc is only found in *P. aeruginosa*, the other T3SS phylogenetic families are distributed across different *Pseudomonas* groups. For example, strains belonging to the *P. fluorescens* group can contain SPI-1, Hrp1 or Rhizobia T3SS.

Although the Hrp1 locus is present in approximately 40% of the *P. fluorescens* genomes (Additional file 6), two distinct sub-families are found in different chromosomal locations [64]. The Hrp1 locus (PSF113_5585-5616) encoded in the F113 chromosome is, perhaps unsurprisingly, closely related to Hrp1 of *P. fluorescens* Q8r1-96 [64], Q2-87 [20], Wood1R [65] and *P. brassicacearum* NFM421 [24]. The predicted T3Es RopAA1, RopB and RopM reported by Mavrodi et al. [64] are also encoded in the genome of F113, Q2-87, Wood1R and NFM421. In addition a putative paralogue of RopAA1, called here RopAA2 (PSF113_3486), is found in all these strains.

In comparison to Hrp1, the SPI-1 phylogenetic family is less abundant in the *Pseudomonas* genome sequences (Additional file 6). Comparative genomics analysis of SPI-1 clusters belonging to strains belonging to the *P. fluorescens* group, revealed that these loci are found in the same chromosomal location, flanked by the genes *nasT* and *pyrD*. In F113, however, the SPI-1 cluster is flanked by *nasT* (PSF113_1777) and *soxR* (PSF113_1805). This is due to genome rearrangement, probably caused by IS3-family transposases (PSF113_1803-1804 and PSF113_4042-4043). Examination of other *P. fluorescens* genomes indicated that the genes *invG*, *iagB* and pseudogene related to *hilA* are present at the same chromosomal location in strains PAMC 25886 [66], Ag1 [67], 14–3 [68], WH6 [69], SBW25 [18], R81 [70], A506, BG33R, SS101 [20], NZ007, NZ052 and PMS117. Interestingly all these strains belong to subgroup V of the *P. fluorescens* group, which suggest that the SPI-1 cluster was lost before speciation of this sub-clade. While the SPI-1 cluster is conserved across strains belonging to the sub-clade composed of UPB0736 [71], CB98818, NCPPB 3146 and NCPPB 2289; its random distribution across the other strains of the *P. fluorescens* group could suggest an acquisition by horizontal gene transfer. Investigation of the average GC content of the SPI-1 clusters did, however, not show any shift in comparison to the average content of the genome examined (Additional file 9). This is not the case with the SPI-1 cluster of two strains belonging to the *P. putida* group, which have an atypical GC content, therefore suggesting horizontal gene transfer acquisition.

#### T6SSs

The Type VI secretion machinery is the product of approximately 15 conserved genes which are generally found together inside a genomic locus [72]. T6SS are widespread in *Proteobacteria*, particularly among gamma-*Proteobacteria* and have been classified into five distinct phylogenetic clusters (from 1 to 5) [73]. However a recent comparative genomic analysis has further divided the cluster 4 into 4A and 4B [74]. *P. aeruginosa* possesses three different loci named HSI-I (cluster 3), II (cluster 1) and III (cluster 4A), which perform different functions. Whereas HSI-I is be involved in inter-bacterial interactions through secretion of Tse1, Tse2 and Tse3 [75-77], HSI-II and III could be linked to virulence towards animals and plants [78,79]. To date, strains belonging to the *P. fluorescens* group possess between one to four T6SSs per genome, which belong to cluster 1 to 4B (Additional files 6 and 10). F113 contains the three most common T6SS phylogenetic clusters, which are related to the HSI loci. Moreover, in this strain the two loci HSI-I (PSF113_5785-5807) and HSI-II (PSF113_5815-PSF113_5830) are located besides each other in a tandem arrangement, which is an uncommon feature of T6SS. In addition to the T6SSs loci, most bacterial strains encode additional *vgrG* and *hcp* genes that encode extracellular structural components of the secretion apparatus, which are encoded elsewhere in the genome [80]. From the eight VgrGs and three Hcps encoded in the F113 genome, five VgrGs and one Hcp proteins are located outside T6SS loci [74].

### Other virulence and niche adaption traits

Different strains of *P. fluorescens* produce a set of secondary metabolites with antifungal and antibacterial properties [81]. These metabolites are important for competition and survival in the rhizosphere and are the basis of biocontrol activities. F113 genome carries gene responsible for siderophores synthesis, including pyoverdine [2] and pyridine-2,6-bis-thiocarboxylic acid [82]. Moreover F113 produces antimicrobial compounds unrelated to siderophores such as hydrogen cyanide [83] and DAPG [4] (Table 2).

**Table 2:** **Biosynthetic clusters involved in secondary metabolism of *P. fluorescens *F113**

**Metabolite**	**Gene involved in biosynthesis**	**Locus number**	**Biological Evidence**	**Reference**
Pyoverdine	*pvdSL; pvdIJKDEONMP; fpvI; pvdA*	PSF113_1749-1750; PSF113_1836-1847; PSF113_1856-1860	Yes	[2]
Pyridine-2,6-bis-thiocarboxylic acid (PDTC)	*pdtCKPELMFGHIJON*	PSF113_2605-2618	No	
2,4 diacetyl-phloroglucinol (DAPG)	*phlIEDBCAFGH*	PSF113_2457-2464	Yes	[4]
Hydrogen cyanide (HCN)	*hcnABC*	PSF113_2367-2369	Yes	[83]
Putative Lankacidin	*lkcABCDEFGJ; pqqFABCDEG*	PSF113_3657-3666; PSF113_5383-5388	No	
PKS-like metabolite		PSF113_3045-3053	No	

**Figure 6:**
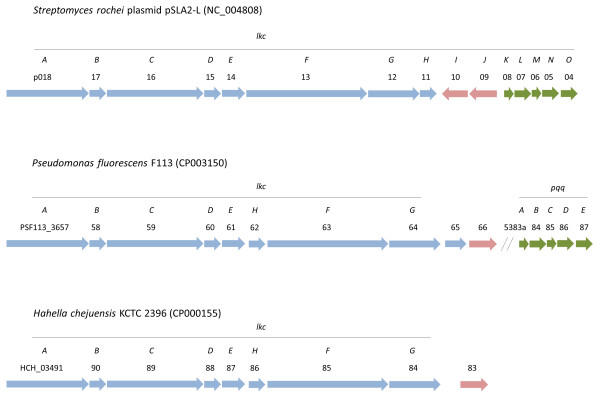
**Comparative genetic analysis of the putative lankacidin biosynthetic cluster.** Genes encoding protein involved in biosynthesis of lankacidin are represented as block arrows showing the direction of their transcription. Blue and pink arrows represent protein-coding genes putatively involved in lankacidin biosynthesis and transport, respectively. Green arrows represent protein-coding genes involved in *pyrroloquinoline quinone* (*PQQ*) synthesis. These genes are not present in the genome of *Hahella chejuensis* KCTC2396.

Besides the genes encoding the enzymes required for the biosynthesis of these compounds, the F113 genome contains a cluster of genes (PSF113_3657- PSF113_3666) that is likely to encode genes for the synthesis of an unknown antibiotic. This 40 Kb cluster encodes four polyketide synthase modules, one dehydratase, one transacylase, one transferase, one isochorismatase and one transport protein (Figure 6). There are not homologous genes to these in the genus *Pseudomonas* and the closest homologues (39-42% homology with coverage over 90% of the sequence) are found in genes responsible of the synthesis of macrolide antibiotics from different *Streptomyces* species, including the lankacidin production cluster of *Streptomyces rochei*[84]. The lankacidin (*lkc*) gene cluster of *S. rochei* is composed of 15 ORFs (*lkcABCDEFGHIJKLMNO*), which encode a polyketide synthase (PKS)/nonribosomal peptide synthetase (NRPS) hybrid gene (*lkcA*), type I PKS genes, and the essential cluster for the lankacidin production, pyrroloquinoline quinone (PQQ) biosynthetic genes (*lkcK-lkcO*) [85,86]. The presence of this cluster in other genomes was therefore investigated by comparative genomic analysis. Besides *Streptomyces rochei* plasmid pSLA2-L and *P. fluorescens* F113 the *lkc* cluster was only found in one additional genome: *Hahella chejuensis* KCTC 2396. In *P. fluorescens* F113, the PQQ genes, which are crucial for lankacidin production in *Streptomyces rochei*[85] are found outside the *lkc* gene cluster [37]. The possible production of this unknown antibiotic is likely to contribute to rhizosphere and soil competence.

It has been shown that *P. fluorescens* strains can kill nematodes [5] and are pathogenic to insects [87]. Preliminary results show that F113 is toxic towards the model nematode *Caenorhabditis elegans* and the protozoan *Acanthamoeba polyphaga.* F113 repels these bacteriovores and/or prevents their growth feeding on this strain (our unpublished data). In addition, F113 kills the model insect *Galleria mellonella*, after 48 h of infection with a dose of 10^8^ bacteria per larvae (our unpublished data). These features could be explained by the production of putative insecticidal toxins (PSF113_0731 and PSF113_0732), which are also found in *Pseudomonas* strains NFM421 and Pf0-1 and in other bacterial species such as *Yersinia enterocolitica*[88]. In addition, a number of other putative toxins and virulence factors are present in *P. fluorescens* F113 genome including 16 predicted hemolysin/haemagglutinins, nine adhesin or agglutination proteins, two RTX toxins, seven Rhs-family proteins and eight YD-repeat-containing proteins.

#### ICE and prophage elements

Analysis of the F113 genome has revealed the presence of one putative integrative conjugative element (ICE) of 175 kb containing 158 ORFs located downstream a tRNA-glycine (PSF113_3287). This putative bacteriophage-derived mobile genetic element is surrounded by *attF* and *attR* sites (TTGGAGCGGGAAACGAGACTCG) and possesses protein-coding genes required for excision and integration (PSF113_3288-3301). The mobilization of the ICE could be mediated by a GI-T4SS (PSF113_3314-3334), which is responsible for the formation of the conjugative pilus and the resulting conjugative transfer [89]. However, it remains to be determined whether this GI-T4SS is functional since a conserved T4SS component is disrupted by a IS66 transposase (PSF113_3325-3327). In addition to protein-coding genes involved in the integration and transfer of this hypothetical ICE, the cargo genes are related to T1SS (PSF113_3303-3305) *dit* cluster (PSF113_3356-3417) and *nos2nor2* (PSF113_3422-3438), described above.

F113 also carry two prophage elements (P1 and P2). Prophage P1 (16,984 bp) is, like the prophages of other *P. fluorescens* strains [90], inserted between two highly conserved genes: *mutS* (PSF113_1167) and *cinA* (PSF113_1173). Prophage P1 closely resembles to prophage Pp1 of *P. fluorescens* SBW25 [18,90] and carries conserved tail synthesis genes of phage CTX but lacks integrase and head morphogenesis genes. Therefore this genomic region probably encodes a bacteriocin belonging to the R-type pyocins. The second prophage of *P. fluorescens* F113 (41,823 bp) is related to prophage 03 of *P. fluorescens* Pf-5 [90]. This genomic region contains protein-coding genes involved in tail assembly and head morphogenesis and therefore could correspond to the genome of a bacteriophage.

## Conclusions

Genome analysis of strain F113 has shown that this bacterium belongs to the *Pseudomonas fluorescens* complex. However, as indicated earlier by Silby et al. [18] and more recently by Loper et al. [20], the taxonomy of *P. fluorescens* requires further study, since significant differences are found in the genomic complement of different strains. Furthermore, the phylogenomic analysis has shown that the *P. fluorescens* group can be subdivided into at least, five subgroups. Indeed, while F113 shared only 35% of its genome with all the other sequenced strains within the *P. fluorescens* group, this value increases to 76% when the F113 genome is compared to the four closest relatives (*P. brassicacearum* Q8r1-96, *P. brassicacearum* NFM421, *P. fluorescens* WoodR1 and *P. fluorescens* Q2-87 genomes). The fact that these strains have common unusual traits such as denitrification, phenotypic variation during rhizosphere colonization [14,91,92] and highly conserved and unique chemotaxis systems, might indicate that these strains belong to the same species. In addition F113 genome contains 344 genes that are not found in Q2-87, Q8r1-96, WoodR1 and NFM421, which encode proteins likely to be involved in the production of an unknown polyketide and in the assembly of a second flagella. These traits could explain in part the excellent rhizocompetence ability of F113 and are currently under study.

## Methods

### Bioinformatic analysis

*Pseudomonas* predicted proteomes were downloaded from the PATRIC ftp server [93] (Additional file 1). Whole-genome based phylogenetic trees were built by using a Composition Vector approach [22,94] using the web server CVTree [95] with a peptide window (k value) equal to 6. Trees were generated by Neighbor joining algorithm using as outgroup *Escherichia coli* K12 when analysing the whole *Pseudomonas* genus and *P. aeruginosa* PAO1 when analysing *P. fluorescens* group. Phylogenetic trees were visualized and exported using MEGA software v5 [96].

Orthologous CDSs in the fifty genomes within the *P. fluorescens* group (Additional file 1) were defined after comparing all-against-all using BLASTP and processed by OrthoMCL pipeline using default settings, match percentage cut-off 50% and an expected value of 1e-5 [25]. Own designed perl scripts and SQL queries were used to filter results. Venn diagram was drawn using R-project language [97] and gplots package [98].

Genome rearrangement between the complete genomes sequences of *P. fluorescens* F113, *P. brassicacearum subsp. brassicacearum* NFM421 and *P. fluorescens* Pf0-1 was assessed by using Mauve software with a window of 1000 nucleotides [99].

The ORFs of *P. aeruginosa* PAO1, *P. fluorescens* Pf-5, *P. putida* KT2440 and *P. syringae* pv. tomato DC3000 were used as queries in BLASTP searches, expected value lower than 1e-5, to identify homologues of TolC (T1SS), XcpR (T2SS), HrcV (T3SS), VirB4 (T4SS), EstA (T5aSS), TpsB (T5bSS), PlpD (T5dSS) and IglA (T6SS) in all *Pseudomonas* predicted proteomes available at the time of analysis. The retrieved sequences were verified using the conserved domain database (CDD) [100]. Protein sequences were aligned using MAFFT [101]. Maximum-likelihood (ML) trees with were built with PhyML [102] using the WAG amino acid substitution model of evolution [103] and four categories of substitution rates. Branch supports were evaluated using the approximate likelihood-ratio test (aLRT) [104]. Phylogenetic trees were visualized and exported using the web-based tool Interactive Tree Of Life (iTol) [105].

### Metabolic and motility assays

Denitrification tests were carried out using *P. fluorescens* F113 and Pf0-1. Inoculi from both strains were obtained from overnight cultures grown in LB medium [106]. Assays were performed in LB medium alone or supplemented either with 20 mM of KNO_3_ or 10 mM of NaNO_2_. Argon gas was used to create anaerobic conditions by purging it into the medium. Presence or absence of NaNO_2_ was checked using NitriVer® 5 Nitrite Reagent Powder Pillows (HACH. Düsseldorf, Germany).

Growth on abietic acid (Sigma. Steinhelm, Germany) was tested by colony counts after 20 days cultivation in minimal medium (0.1 g/L NaCl, 0.1 g/L MgSO_4_ 7 H_2_O, 1 g/L K_2_HPO_4_, 0.5 g/L KH_2_PO_4_, 1 g/L NH_4_SO_4_) supplemented with 1 ml/L PAS salts (19.5 g/L MgSO_4_, 5 g/L MnSO_4_ H_2_O, 1 g/L FeSO_4_ 7 H_2_O and 0.3 g/L CaCl H_2_O) and with 0.01% abietic acid. Cells from strains F113 and Pf0-1 were conditioned for 10 days in this medium. Approximately 10^4^ cells from these cultures were inoculated in the same medium and incubated 20 days at 28°C. Dilutions of these cultures were plated on SA medium and colonies were counted.

SA medium plates containing 0.3% purified agar were used to test swimming abilities. The cells from exponentially growing cultures were inoculated into the plates using a toothpick. Swimming haloes were checked after 18 h of inoculation. Every assay was performed three times with three replicates each time.

ACC deaminase activity was measured by determining the production of α-ketobutyrate from 1-amino-1-cyclopropane carboxylic acid (ACC). The strain was cultured at 30°C overnight in 4.75 ml *Pseudomonas* minimal media broth supplemented with 0.15% glucose, 1% sodium citrate and 0.25 ml of 0.5 M ACC. The cells were harvested by centrifugation (2 min at 13,000 rpms) and resuspended in 0.2 ml 0.1 M Tris–HCl (pH 7.5). 10 μl of toluene was added to the cell suspension and mixed. 50 μL of the toluenised cells were transferred into a clean dry eppendroff tube. 5 μL of 0.5 M ACC was added into the tube and incubated for 30 min at 30°C. 500 μL of 0.56 M HCl was added into the tube and vortexed. The cell debris was removed by centrifugation and 500 μL of the supernatant was transferred to a clean tube. 400 μL of 0.56 M HCl and 150 μL of 2% 2–4 dinitrophenylhydrazine solution were added and the mixture was incubated at 30°C for 30 mins. 1 ml of 2 M NaOH was then added to the samples. The absorbance of each sample was measured at 540 nm. α-ketobutyrate concentrations were calculated from a standard curve created using α-ketobutyrate standards.

## Competing interests

The authors declare that they have no competing interests.

## Authors’ contributions

MR-N and MB performed the, phylogenomic analysis and bioinformatic analysis and participated in drafting the Ms. JM and JAM contributed to the bioinformatic analysis. KG carried out studies on the expression of ACC deaminase. FM-G, EB, AN and MR-N analyzed motility genes and performed motility experiments. CM and MR-N analyzed the denitrification and chemotaxis genes and performed experiments on these two traits. MS-C analyzed genes implicated in virulence and performed growth experiments on abietic acid and on interaction with invertebrates. JM, DD, FOG, MM and RR were involved in the conception of the study, participated in its design and coordination, supervised work and wrote the Ms. All authors read and approved the final manuscript.

## Supplementary Material

Additional file 1 **Characteristics of sequenced genomes from the *P. fluorescens* group (as of 20-11-2012).** Click here for file

Additional file 2 **Physical map of the *Pseudomonas fluorescens* F113 genome.** Arrows in the outer circles show CDS (in green), tRNA (in red) and rRNA (in yellow). Gene clusters described in the article are highlighted in the map. Center circle shows G/C% distribution. Click here for file

Additional file 3 **List of F113 CDSs that are not present or are below the threshold to be considered as orthologous in other strains belonging to the * P. fluorescens * Subgroup I.** Closest homologues are included in the Table. Click here for file

Additional file 4 **Distribution of denitrification pathway in strains belonging to the *P. fluorescens* group.** Click here for file

Additional file 5 **Distribution of chemotaxis systems in strains belonging to the*P. fluorescens*group.** Click here for file

Additional file 6 **Repertoire of T2SSs, T3SSs and T6SSs in the *Pseudomonas* genus.** Click here for file

Additional file 7 **Phylogenetic distribution of T2SSs within *Pseudomonas* species.** A distance tree (Maximum-Likelihood) was calculated from 200 XcpR-like proteins of *Pseudomonas* spp. T2SSs of *Pseudomonas* spp. can be divided in two distinct phylogenetic clusters: Xcp and Hxc highlighted in red and blue, respectively. The XcpR-like proteins related to the Hpl cluster are used as out group. Genes of *P. fluorescens* F113 encoding potential T2SSs are represented as block arrows showing the direction of their transcription. Numbers represented the PSF113 locus IDs. Black arrows represented genes encoding the T2S machinery, whereas grey arrows represented gene encoding protein with unknown function. White arrows represented genes encoding putative T2SS-substrates. Click here for file

Additional file 8 **Phylogenetic distribution of T3SSs within *Pseudomonas* species.** A distance tree (Maximum-Likelihood) was calculated from 146 HrcV-like proteins of *Pseudomonas* spp. Green, orange, and blue labels indicate HrcV-like proteins related to Hrp1, SPI-1, Rhizobiales, and Ysc systems, respectively. Genes of *P. fluorescens* F113 encoding T3SSs are represented as block arrows showing the direction of their transcription. Numbers represented the PSF113 locus IDs. Black arrows represented genes encoding the T3S machinery, whereas grey arrows represented gene encoding protein with unknown function. White arrows represented genes encoding putative T3SS-substrates and chaperones. Click here for file

Additional file 9 **GC content analysis of the SPI-1 clusters encoded in different strains belonging to the *Pseudomonas* genus.** Asterisks indicate that the SPI-1 cluster is located on two different scaffolds. Click here for file

Additional file 10 **Phylogenetic distribution of T6SSs within *Pseudomonas* species.** A distance tree (Maximum-Likelihood) was calculated from 345 IglA proteins of *Pseudomonas* spp. T6SSs of *Pseudomonas* spp. can be divided into five mains clusters (1, 2, 3, 4A and 4B) highlighted in red, yellow, green, blue and purple, respectively. Genes of *P. fluorescens* F113 encoding T6SSs are represented as block arrows showing the direction of their transcription. Numbers represented the PSF113 locus IDs. Black arrows represented genes encoding the T6S machinery, whereas grey arrows represented gene encoding protein with unknown function. Click here for file
