# Supplementary material for: Genome sequence reveals that Pseudomonas fluorescens F113 possesses a large and diverse array of systems for rhizosphere function and host interaction
Source: BMC Genomics. 2013 Jan 25;14:54. doi: 10.1186/1471-2164-14-54 (PMC3570484; doi:10.1186/1471-2164-14-54)
Supplement: Additional file 1 — Characteristics of sequenced genomes from the P. fluorescens group (as of 20-11-2012). [file 1471-2164-14-54-S1.pdf]

Strain	Type	INSDC	Size (Mb)	GC%	CDS	rRNA	tRNA	Other RNA	Reference
***P. brassicacearum* NFM421**	Complete	CP002585.1	6.84	60.8	6095	16	65	-	[1]
***P. brassicacearum* Q8r1-96**	Draft (5)	CM001512.1	6.60	61.0	5715	16	65	20	[2]
***Pseudomonas chlororaphis subsp. aureofaciens* 30-84**	Draft(13)	CM001559.1	6.66	62.9	5848	19	74	19	[2]
***P. chlororaphis* GP72**	Draft (347)	AHAY00000000.1	6.63	63.1	6107	-	56	-	[3]
***P. chlororaphis* O6**	Draft (31)	CM001490.1	6.98	62.9	6,223	-	-	-	[2]
***P. extremaustralis* 14-3 substr 14-3b**	Draft (113)	AHIP00000000.1	6.59	60.7	5934	13	49	-	[4]
***P. fluorescens* A506**	Complete	CP003041.1	5.96	60.0	5627	19	69	17	unpublished
***P. fluorescens* F113**	Complete	CP003150.1	6.85	60.8	5862	16	66	9	[5]
***P. fluorescens* NCIMB 11764**	Draft(831)	CM001560.1	6.69	58.6	6011	4	30	-	[6]
***P. fluorescens* NZ007**	Draft(809)	AKBR00000000.1	6.47	60	5730	7	47	-	unpublished
***P. fluorescens* NZ011**	Draft(973)	AJXJ00000000.1	6.8	58.5	5923	6	53	-	unpublished
***P. fluorescens* NZ052**	Draft(440)	AJXH00000000.1	6.82	60.1	6078	5	52	-	unpublished
***P. fluorescens* NZ17**	Draft(1032)	AJXF00000000.1	6.8	63.2	6311	3	48	-	unpublished
***P. fluorescens* Pf0-1**	Complete	CP000094.2	6.85	60.5	5722	19	73	3	[7]
***P. fluorescens* Q2-87**	Draft(2)	AGBM00000000.1	6.36	60.6	5701	19	68		[2]
***P. fluorescens* R124**	Draft(77)	ALYL01000000	6.25	60.3	5570	-	-	-	unpublished
***P. fluorescens* SBW25**	Complete	AM181176.4	6.72	60.5	5921	16	66	15	[7]
***P. fluorescens* SS101**	Complete	CM001513.1	6.18	60.0	5372	19	68	17	unpublished
***P. fluorescens* Wayne1**	Draft (90)	CADX01000000	6.86	-	6228	-	52	-	[8]
***P. fluorescens* WH6**	Draft(53)	CM001025.1	6.27	60.5	5833	4	52	-	[9]
***P. fluorescens* Wood1R**	Draft (1437)	CAFF01000000	6.68	-	5897	-	30	-	[8]
***P. mandelii* JR-1**	Draft (96)	AJFM00000000.1	7.10	57.9	6374	5	54	-	[10]
***P. protegens* Pf-5**	Complete	CP000076.1	7.07	63.3	6108	16	71	17	[11]
***P. tolaasii* NCPPB 2192**	Draft(13915)	AJXK00000000	14.37	60.2	12781	10	63	-	unpublished
***P. tolaasii* PMS117**	Draft(357)	AJXG00000000	7.0	60.2	6178	3	52	-	unpublished
***Pseudomonas sp* Ag1**	Draft(113)	AKVH00000000	7.25	-	6482	4	48	-	[12]
***P. synxantha* BG33R**	Complete	CM001514.1	6.30	59.7	5509	19	68	18	[2]
***Pseudomonas sp* GM102**	Draft(159)	AKJB00000000	6.65	-	6001	3	58	12	[13]
***Pseudomonas sp* GM16**	Draft(128)	AKJV00000000	6.55	-	5867	2	64	13	[13]
***Pseudomonas sp* GM17**	Draft(280)	AKJU00000000	6.78	-	6085	3	56	13	[13]
***Pseudomonas sp* GM18**	Draft(140)	AKJT00000000	6.29	-	5682	2	61	12	[13]
***Pseudomonas sp* GM21**	Draft(210)	AKJS00000000	6.60	-	6031	3	53	11	[13]
***Pseudomonas sp* GM24**	Draft(399)	AKJR00000000	6.51	-	5828	7	54	12	[13]

Strain	Type	INSDC	Size (Mb)	GC%	CDS	rRNA	tRNA	Other RNA	Reference
***Pseudomonas sp* GM25**	Draft(91)	AKJQ00000000	6.35	-	5684	2	51	11	[13]
***Pseudomonas sp* GM30**	Draft(180)	AKJP00000000	6.14	-	5588	2	58	11	[13]
***Pseudomonas sp* GM33**	Draft(205)	AKJO00000000	6.72	-	6061	2	49	11	[13]
***Pseudomonas sp* GM41(2012)**	Draft(164)	AKJN00000000	6.61	-	6012	3	57	12	[13]
***Pseudomonas sp* GM48**	Draft(200)	AKJM00000000	6.44	-	5845	3	47	11	[13]
***Pseudomonas sp* GM49**	Draft(345)	AKJL00000000	6.58	-	6227	3	57	13	[13]
***Pseudomonas sp* GM50**	Draft(155)	AKJK00000000	6.69	-	6033	3	57	11	[13]
***Pseudomonas sp* GM55**	Draft(163)	AKJJ00000000	6.48	-	5949	7	50	2	[13]
***Pseudomonas sp* GM60**	Draft(181)	AKJI00000000	6.42	-	5884	3	54	2	[13]
***Pseudomonas sp* GM67**	Draft(183)	AKJH00000000	6.50	-	5966	2	57	2	[13]
***Pseudomonas sp* GM74**	Draft(180)	AKJG00000000	6.10	-	5531	5	54	2	[13]
***Pseudomonas sp* GM78**	Draft(235)	AKJF00000000	7.28	-	6681	2	57	1	[13]
***Pseudomonas sp* GM79**	Draft(126)	AKJE00000000	6.70	-	6026	2	58	1	[13]
***Pseudomonas sp* GM80**	Draft(282)	AKJD00000000	6.78	-	6183	3	59	2	[13]
***Pseudomonas sp* PAMC 25886**	Draft (95)	AHHC00000000.1	7.02	59.9	5830	-	50	-	[14]
***Pseudomonas sp* R62**	Draft (991)	AHZM00000000.1	6.32	59.5	5354	-	-	-	[15]
***Pseudomonas sp* R81**	Draft (151)	AHZN01000000.1	6.22	61.7	5602	-	-	-	[15]

Numbers presented in parentheses indicate the number of contigs.

REFERENCES

1. Ortet P, Barakat M, Lalaouna D, Fochesato S, Barbe V, Vacherie B, Santaella C, Heulin T, Achouak W: **Complete genome sequence of a beneficial plant root-associated bacterium, *Pseudomonas brassicacearum***. *J Bacteriol* 2011, **193**(12):3146.
2. Loper JE, Hassan KA, Mavrodi DV, Davis EW, 2nd, Lim CK, Shaffer BT, Elbourne LD, Stockwell VO, Hartney SL, Breakwell K *et al*: **Comparative genomics of plant-associated *Pseudomonas* spp.: insights into diversity and inheritance of traits involved in multitrophic interactions**. *PLoS Genetics* 2012, **8**(7):e1002784.
3. Shen X, Chen M, Hu H, Wang W, Peng H, Xu P, Zhang X: **Genome sequence of *Pseudomonas chlororaphis* GP72, a root-colonizing biocontrol strain**. *J Bacteriol* 2012, **194**(5):1269-1270.
4. Tribelli PM, Raiger Iustman LJ, Catone MV, Di Martino C, Revale S, Mendez BS, Lopez NI: **Genome sequence of the polyhydroxybutyrate producer *Pseudomonas extremaustralis*, a highly stress-resistant Antarctic bacterium**. *J Bacteriol* 2012, **194**(9):2381-2382.
5. Redondo-Nieto M, Barret M, Morrisey JP, Germaine K, Martinez-Granero F, Barahona E, Navazo A, Sanchez-Contreras M, Moynihan JA, Giddens SR *et al*: **Genome sequence of the biocontrol strain *Pseudomonas fluorescens* F113**. *J Bacteriol* 2012, **194**(5):1273-1274.
6. Vilo CA, Benedik MJ, Kunz DA, Dong Q: **Draft Genome Sequence of the Cyanide-Utilizing Bacterium *Pseudomonas fluorescens* Strain NCIMB 11764**. *J Bacteriol* 2012, **194**(23):6618-6619.
7. Silby M, Cerdeno-Tarraga A, Vernikos G, Giddens S, Jackson R, Preston G, Zhang X-X, Moon C, Gehrig S, Godfrey S *et al*: **Genomic and genetic analyses of diversity and plant interactions of *Pseudomonas fluorescens***. *Genome Biology* 2009, **10**(5):R51.
8. Rong X, Gurel FB, Meulia T, McSpadden Gardener BB: **Draft genome sequences of the *Pseudomonas fluorescens* biocontrol strains Wayne1R and Wood1R**. *J Bacteriol* 2012, **194**(3):724-725.
9. Kimbrel JA, Givan SA, Halgren AB, Creason AL, Mills DI, Banowetz GM, Armstrong DJ, Chang JH: **An improved, high-quality draft genome sequence of the Germination-Arrest Factor-producing *Pseudomonas fluorescens* WH6**. *BMC Genomics* 2010, **11**:522.
10. Jang SH, Kim J, Hong S, Lee C: **Genome sequence of cold-adapted *Pseudomonas mandelii* strain JR-1**. *J Bacteriol* 2012, **194**(12):3263.
11. Paulsen IT, Press CM, Ravel J, Kobayashi DY, Myers GS, Mavrodi DV, DeBoy RT, Seshadri R, Ren Q, Madupu R *et al*: **Complete genome sequence of the plant commensal *Pseudomonas fluorescens* Pf-5**. *Nat Biotechnol* 2005, **23**(7):873-878.
12. Wang Y, Gilbreath TM, 3rd, Kukutla P, Yan G, Xu J: **Dynamic gut microbiome across life history of the malaria mosquito *Anopheles gambiae* in Kenya**. *PLoS One* 2011, **6**(9):e24767.
13. Brown SD, Utturkar SM, Klingeman DM, Johnson CM, Martin SL, Land ML, Lu TY, Schadt CW, Doktycz MJ, Pelletier DA: **Twenty-one genome sequences from *Pseudomonas* species and 19 genome sequences from diverse bacteria isolated from the rhizosphere and endosphere of *Populus deltoides***. *J Bacteriol* 2012, **194**(21):5991-5993.
14. Shin SC, Kim SJ, Hong SG, Ahn do H, Lee YM, Lee H, Lee J, Park H: **Genome sequence of *Pseudomonas* sp. strain PAMC 25886, isolated from alpine glacial cryoconite**. *J Bacteriol* 2012, **194**(7):1844.
15. Mathimaran N, Srivastava R, Wiemken A, Sharma AK, Boller T: **Genome sequences of two plant growth-promoting fluorescent *Pseudomonas* strains, R62 andR r81**. *J Bacteriol* 2012, **194**(12):3272-3273.
