# Supplementary figures and images for: Genome sequence reveals that Pseudomonas fluorescens F113 possesses a large and diverse array of systems for rhizosphere function and host interaction
Source: BMC Genomics. 2013 Jan 25;14:54. doi: 10.1186/1471-2164-14-54 (PMC3570484; doi:10.1186/1471-2164-14-54)

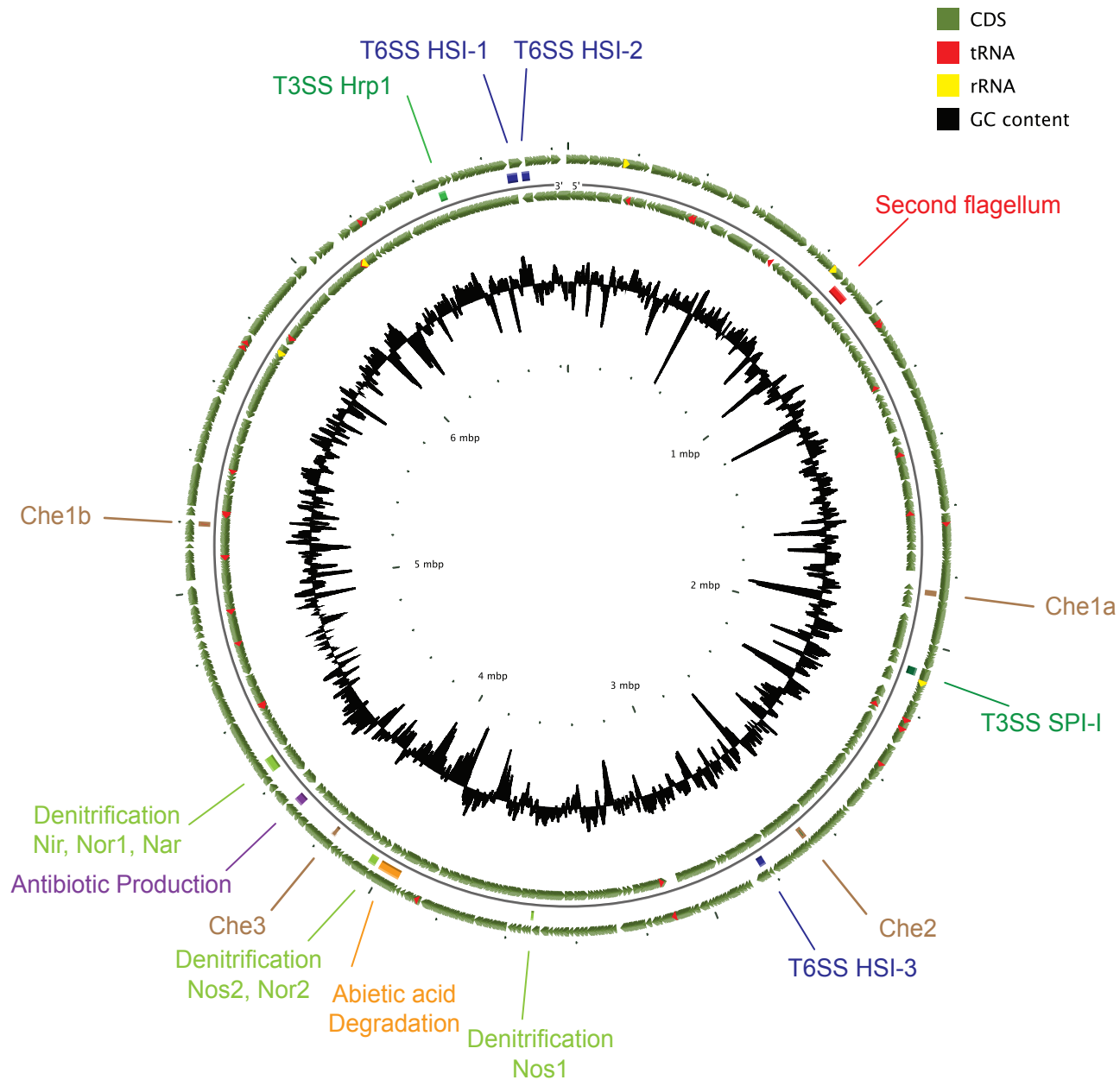

Supplement: Additional file 2 — Physical map of the Pseudomonas fluorescens F113 genome. Arrows in the outer circles show CDS (in green), tRNA (in red) and rRNA (in yellow). Gene clusters described in the article are highlighted in the map. Center circle shows G/C% distribution. [file 1471-2164-14-54-S2.pdf]

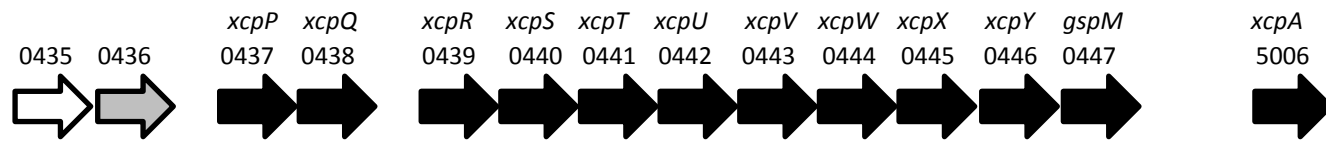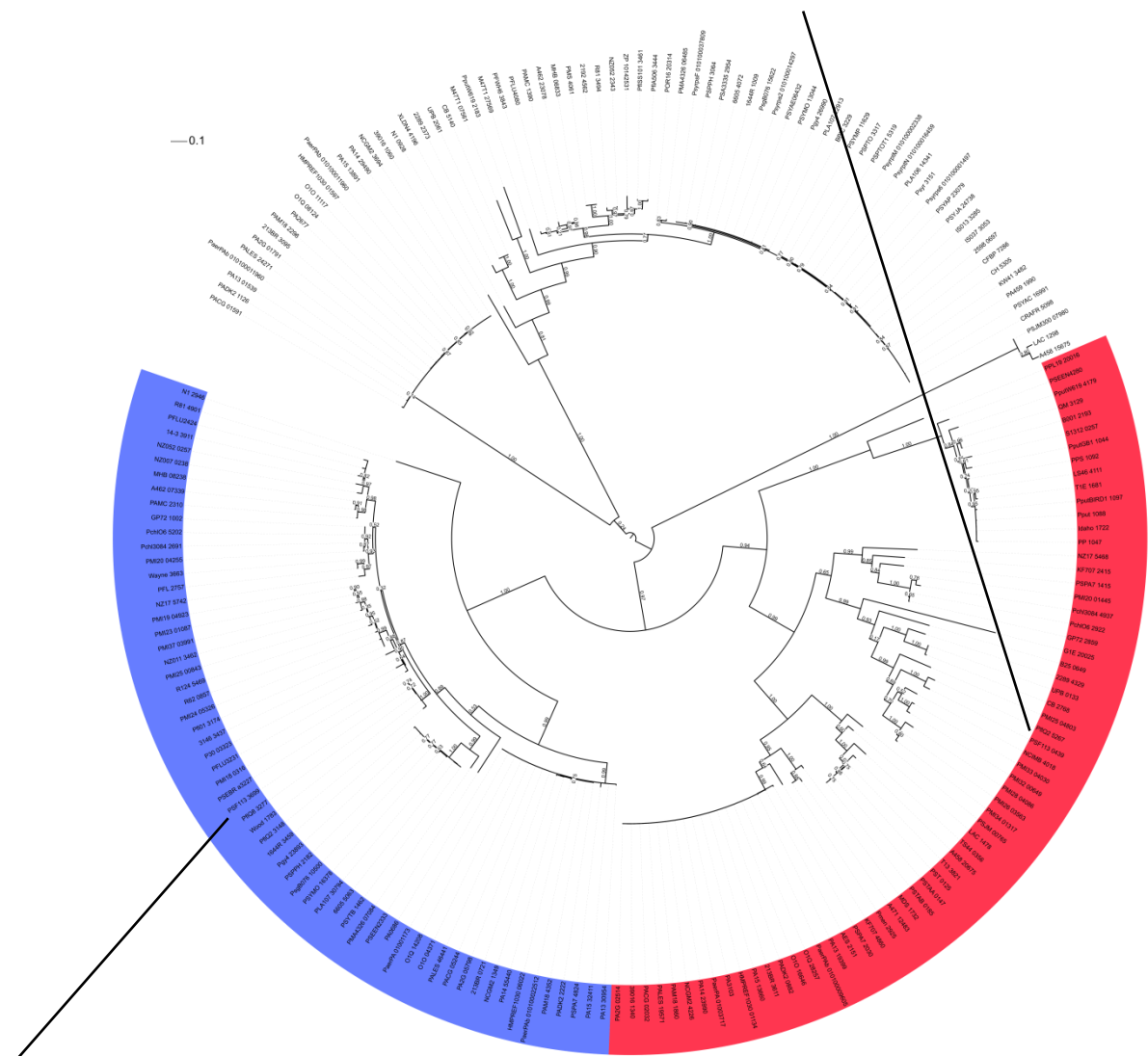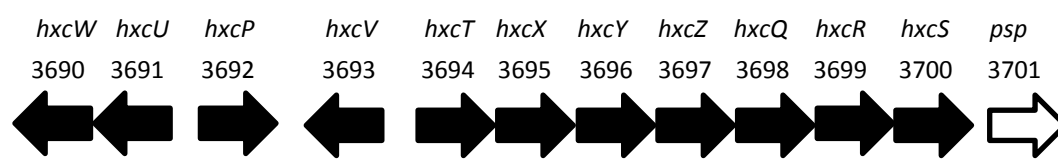

Supplement: Additional file 7 — Phylogenetic distribution of T2SSs within Pseudomonas species. A distance tree (Maximum-Likelihood) was calculated from 200 XcpR-like proteins of Pseudomonas spp. T2SSs of Pseudomonas spp. can be divided in two distinct phylogenetic clusters: Xcp and Hxc highlighted in red and blue, respectively. The XcpR-like proteins related to the Hpl cluster are used as out group. Genes of P. fluorescens F113 encoding potential T2SSs are represented as block arrows showing the direction of their transcription. Numbers represented the PSF113 locus IDs. Black arrows represented genes encoding the T2S machinery, whereas grey arrows represented gene encoding protein with unknown function. White arrows represented genes encoding putative T2SS-substrates. [file 1471-2164-14-54-S7.pdf]

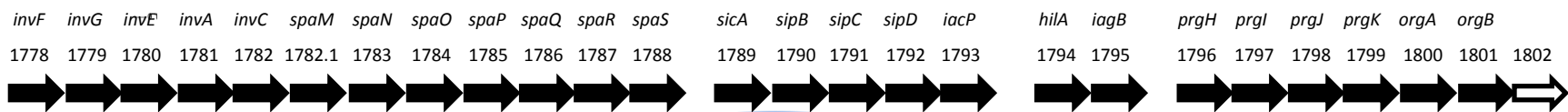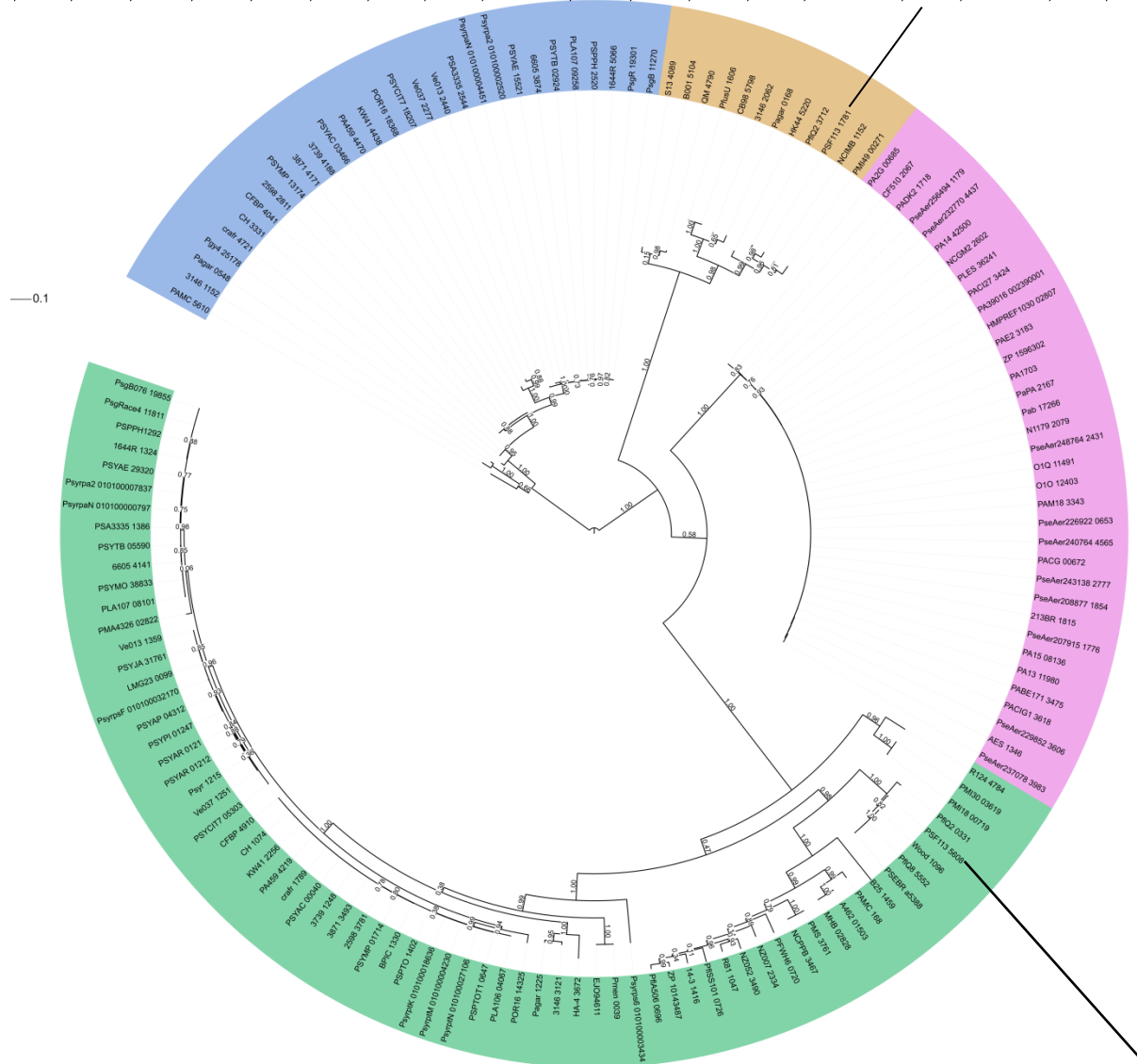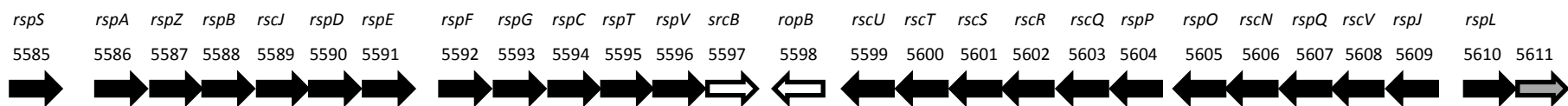

Supplement: Additional file 8 — Phylogenetic distribution of T3SSs within Pseudomonas species. A distance tree (Maximum-Likelihood) was calculated from 146 HrcV-like proteins of Pseudomonas spp. Green, orange, and blue labels indicate HrcV-like proteins related to Hrp1, SPI-1, Rhizobiales, and Ysc systems, respectively. Genes of P. fluorescens F113 encoding T3SSs are represented as block arrows showing the direction of their transcription. Numbers represented the PSF113 locus IDs. Black arrows represented genes encoding the T3S machinery, whereas grey arrows represented gene encoding protein with unknown function. White arrows represented genes encoding putative T3SS-substrates and chaperones. [file 1471-2164-14-54-S8.pdf]

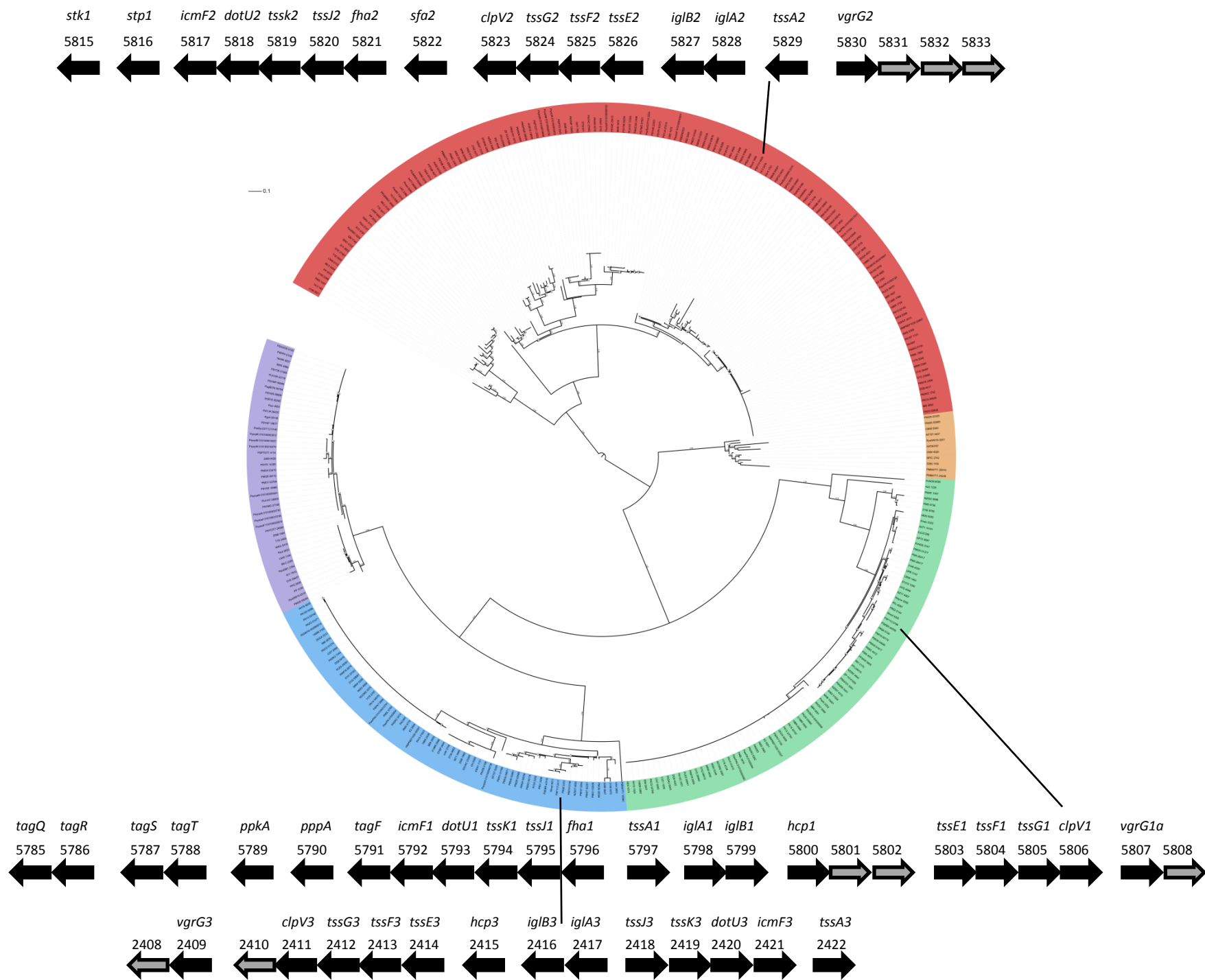

Supplement: Additional file 10 — Phylogenetic distribution of T6SSs within Pseudomonas species. A distance tree (Maximum-Likelihood) was calculated from 345 IglA proteins of Pseudomonas spp. T6SSs of Pseudomonas spp. can be divided into five mains clusters (1, 2, 3, 4A and 4B) highlighted in red, yellow, green, blue and purple, respectively. Genes of P. fluorescens F113 encoding T6SSs are represented as block arrows showing the direction of their transcription. Numbers represented the PSF113 locus IDs. Black arrows represented genes encoding the T6S machinery, whereas grey arrows represented gene encoding protein with unknown function. [file 1471-2164-14-54-S10.pdf]
