# Supplementary material for: Genome sequence reveals that Pseudomonas fluorescens F113 possesses a large and diverse array of systems for rhizosphere function and host interaction
Source: BMC Genomics. 2013 Jan 25;14:54. doi: 10.1186/1471-2164-14-54 (PMC3570484; doi:10.1186/1471-2164-14-54)
Supplement: Additional file 3 — List of F113 CDSs that are not present or are below the threshold to be considered as orthologous in other strains belonging to the P. fluorescens Subgroup I. Closest homologues are included in the Table. [file 1471-2164-14-54-S3.pdf]

F113 Locus	Type	Strand	Coordinates	Length (aa)	Gene	Function	Organism	Hit ACC	E-Value	Similarity	Alignment length
**PSF113_0319**	CDS	+	382794..382997	67		YafQ toxin protein	Pseudomonas sp. GM30	ZP_10679031.1	5E-36	54	67
**PSF113_0322**	CDS	-	384297..385454	385		hypothetical protein	Pseudomonas sp. GM21	ZP_10700790.1	0	346	385
**PSF113_0453**	CDS	-	539485..540699	404		Major facilitator family transporter	Pseudomonas sp. GM84	ZP_10605236.1	0	321	404
**PSF113_0454**	CDS	-	543300..543497	65		hypothetical protein	Pseudomonas sp. GM48	ZP_10664277.1	2E-28	54	64
**PSF113_0738**	CDS	-	894478..895422	314	motD	MotD	Pseudomonas extremaustralis 14-3 substr. 14-3b	ZP_10438812.1	1E-107	174	261
**PSF113_0739**	CDS	-	895419..896243	274	motC	Flagellar motor rotation protein MotA	Pseudomonas extremaustralis 14-3 substr. 14-3b	ZP_10438813.1	2E-163	234	274
**PSF113_0740**	CDS	+	896563..897615	350	fliC2	Flagellar biosynthesis protein FliC	Pseudomonas extremaustralis 14-3 substr. 14-3b	ZP_10438814.1	0	327	350
**PSF113_0741**	CDS	+	897798..901226	1142		TPR domain protein,putative component of TonB system	Bordetella petrii DSM 12804	YP_001631626.1	0	512	1111
**PSF113_0743**	CDS	-	902625..903317	230	fliA2	RNA polymerase sigma factor for flagellar operon	Pseudomonas extremaustralis 14-3 substr. 14-3b	ZP_10439966.1	3E-141	197	230
**PSF113_0745**	CDS	-	903997..906291	764	flhF2	Flagellar biosynthesis protein FlhF	Azotobacter vinelandii DJ	YP_002799937.1	0	380	753
**PSF113_0747**	CDS	-	908378..909547	389	flhB	Flagellar biosynthesis protein FlhB	Pseudomonas extremaustralis 14-3 substr. 14-3b	ZP_10439969.1	0	332	386
**PSF113_0748**	CDS	-	909651..910553	300	motB2	Flagellar motor rotation protein MotB	Pseudomonas extremaustralis 14-3 substr. 14-3b	ZP_10439970.1	5E-175	242	280
**PSF113_0749**	CDS	-	910550..911434	294	motA2	Flagellar motor rotation protein MotA	Enterobacteriaceae bacterium 9_2_54FAA	ZP_07950070.1	8E-118	173	281
**PSF113_0750**	CDS	-	911674..912240	188	flhC	Flagellar transcriptional activator FlhC	Pseudomonas extremaustralis 14-3 substr. 14-3b	ZP_10439972.1	9E-126	175	187
**PSF113_0751**	CDS	-	912244..912486	80	flhD	FlhD	Pseudomonas extremaustralis 14-3 substr. 14-3b	ZP_10439973.1	9E-46	73	80
**PSF113_0753**	CDS	-	913278..913592	104	fliS2	FliS2	Pseudomonas extremaustralis 14-3 substr. 14-3b	ZP_10439975.1	5E-55	90	104
**PSF113_0754**	CDS	-	913704..915113	469	fliD2	Flagellar hook-associated protein FliD	Pseudomonas extremaustralis 14-3 substr. 14-3b	ZP_10439976.1	0	376	453
**PSF113_0755**	CDS	-	915302..915622	106	fliE2	FliE2	Pseudomonas extremaustralis 14-3 substr. 14-3b	ZP_10439977.1	2E-49	84	104
**PSF113_0756**	CDS	+	915882..917606	574	fliF2	Flagellar M-ring protein FliF	Pseudomonas extremaustralis 14-3 substr. 14-3b	ZP_10439978.1	0	465	577
**PSF113_0757**	CDS	+	917615..918601	328	fliG2	Flagellar motor switch protein FliG	Pseudomonas extremaustralis 14-3 substr. 14-3b	ZP_10439979.1	0	303	328
**PSF113_0758**	CDS	+	918594..919316	240	fliH2	FliH2	Pseudomonas extremaustralis 14-3 substr. 14-3b	ZP_10439980.1	5E-112	164	219
**PSF113_0760**	CDS	+	920733..921203	156	fliJ2	FliJ2	Pseudomonas extremaustralis 14-3 substr. 14-3b	ZP_10439982.1	5E-81	125	155
**PSF113_0761**	CDS	+	921659..922600	313	fliK2	FliK2	Polaromonas sp. CF318	ZP_10562011.1	2E-21	51	98
**PSF113_0762**	CDS	+	922724..923197	157	fliL2	FliL2	Pseudomonas extremaustralis 14-3 substr. 14-3b	ZP_10439984.1	1E-54	103	156
**PSF113_0763**	CDS	+	923201..924253	350	fliM2	Flagellar motor switch protein FliM	Azotobacter vinelandii DJ	YP_002799578.1	0	270	350
**PSF113_0764**	CDS	+	924246..924716	156	fliN2	FliN2	Pseudomonas extremaustralis 14-3 substr. 14-3b	ZP_10439986.1	1E-65	119	159
**PSF113_0765**	CDS	+	924770..925135	121	fliO2	FliO2	Pseudomonas extremaustralis 14-3 substr. 14-3b	ZP_10439987.1	2E-35	75	112
**PSF113_0766**	CDS	+	925132..925902	256	fliP2	Flagellar biosynthesis protein FliP	Pseudomonas extremaustralis 14-3 substr. 14-3b	ZP_10439988.1	8E-135	210	226
**PSF113_0767**	CDS	+	925928..926197	89	fliQ2	FliQ2	Pseudomonas extremaustralis 14-3 substr. 14-3b	ZP_10439989.1	3E-52	84	89

F113 Locus	Type	Strand	Coordinates	Length (aa)	Gene	Function	Organism	Hit ACC	E-Value	Similarity	Alignment length
**PSF113_0768**	CDS	+	926203..926991	262	fliR2	FliR2	Pseudomonas extremaustralis 14-3 substr. 14-3b	ZP_10439990.1	4E-150	235	262
**PSF113_0769**	CDS	-	927134..928330	398	flgL2	FlgL2	Pseudomonas extremaustralis 14-3 substr. 14-3b	ZP_10439991.1	0	352	398
**PSF113_0770**	CDS	-	928357..929967	536	flgK2	FlgK2	Pseudomonas extremaustralis 14-3 substr. 14-3b	ZP_10439992.1	0	462	535
**PSF113_0771**	CDS	-	930110..931072	320	flgJ2	FlgJ2	Pseudomonas extremaustralis 14-3 substr. 14-3b	ZP_10439993.1	2E-170	240	303
**PSF113_0772**	CDS	-	931072..932226	384	flgI2	Flagellar P-ring protein FlgI	Azotobacter vinelandii DJ	YP_002799569.1	0	262	350
**PSF113_0773**	CDS	-	932238..932882	214	flgH2	Flagellar L-ring protein FlgH	Pseudomonas extremaustralis 14-3 substr. 14-3b	ZP_10439995.1	2E-123	184	213
**PSF113_0774**	CDS	-	932942..933724	260	flgG2	Flagellar basal-body rod protein FlgG	Pseudomonas extremaustralis 14-3 substr. 14-3b	ZP_10439996.1	4E-180	247	260
**PSF113_0775**	CDS	-	933756..934502	248	flgF2	Flagellar basal-body rod protein FlgF	Pseudomonas extremaustralis 14-3 substr. 14-3b	ZP_10439997.1	2E-151	216	241
**PSF113_0776**	CDS	-	934515..935663	382	flgE2	Flagellar hook protein FlgE	Pseudomonas extremaustralis 14-3 substr. 14-3b	ZP_10439998.1	0	343	382
**PSF113_0777**	CDS	-	935746..936423	225	flgD2	FlgD2	Pseudomonas extremaustralis 14-3 substr. 14-3b	ZP_10439999.1	3E-126	204	225
**PSF113_0778**	CDS	-	936427..936834	135	flgC2	Flagellar basal-body rod protein FlgC	Pseudomonas extremaustralis 14-3 substr. 14-3b	ZP_10440000.1	5E-89	130	135
**PSF113_0779**	CDS	-	936850..937263	137	flgB2	FlgB2	Pseudomonas extremaustralis 14-3 substr. 14-3b	ZP_10440001.1	8E-82	124	137
**PSF113_0780**	CDS	+	937426..938136	236	flgA2	FlgA2	Pseudomonas extremaustralis 14-3 substr. 14-3b	ZP_10440002.1	1E-99	149	207
**PSF113_0781**	CDS	+	938322..938519	65	flgM2	FlgM2	Pseudomonas extremaustralis 14-3 substr. 14-3b	ZP_10440003.1	1E-15	39	59
**PSF113_0782**	CDS	+	938559..938990	143	flgN2	FlgN2	Pseudomonas extremaustralis 14-3 substr. 14-3b	ZP_10440004.1	4E-65	104	137
**PSF113_0810**	CDS	-	980757..981017	86	higB2	HigB	Delftia acidovorans SPH-1	YP_001562241.1	2E-31	56	79
**PSF113_0811**	CDS	-	981310..982338	342		transcriptional regulator, AraC family	Pseudomonas putida S16	YP_004700345.1	1E-156	213	246
**PSF113_0812**	CDS	+	982497..983366	289		putative solute-binding component of ABC transporter	Pseudomonas putida S16	YP_004700348.1	0	255	290
**PSF113_0816**	CDS	+	985574..987001	475		Aspartyl-tRNA(Asn)amidotransferase subunit A @ Glutamyl-tRNA(Gln) amidotransferase subunit A	Acidovorax sp. CF316	ZP_10391513.1	0	267	457
**PSF113_0819**	CDS	-	989330..990913	527		Phytoene dehydrogenase-related protein	Agrobacterium radiobacter K84	YP_002546768.1	0	326	526
**PSF113_0822**	CDS	+	992664..993443	259		3-oxoacyl-[acyl-carrier protein] reductase (EC 1.1.1.100)	Burkholderia sp. H160	ZP_03263858.1	3E-125	177	257
**PSF113_0824**	CDS	+	994800..995816	338		Possible gluconolactonase	Pseudomonas fluorescens SBW25	YP_002871579.1	0	270	338
**PSF113_0825**	CDS	+	995845..997128	427		Histidinol dehydrogenase(EC 1.1.1.23)	Methylobacterium nodulans ORS 2060	YP_002499446.1	0	284	425
**PSF113_0826**	CDS	+	997128..997880	250		3-oxoacyl-[acyl-carrier protein] reductase (EC 1.1.1.100)	Runella slithyformis DSM 19594	YP_004654053.1	4E-63	117	258
**PSF113_0827**	CDS	+	997891..998322	143		Glyoxalase/bleomycin resistance protein/dioxygenase	Burkholderia sp. H160	ZP_03263855.1	2E-47	73	128
**PSF113_0831**	CDS	+	1001695..1003137	480		Carbohydrate-selective porin	Pseudomonas fluorescens SBW25	YP_002871586.1	0	303	457
**PSF113_0832**	CDS	+	1003152..1003895	247		3-oxoacyl-[acyl-carrier protein] reductase	Burkholderia sp. H160	ZP_03263843.1	4E-79	130	244
**PSF113_0992**	CDS	-	1194081..1194440	119		Mobile element protein	Pseudomonas syringae pv. japonica str. M301072	ZP_16685788.1	3E-79	117	119
**PSF113_0994**	CDS	-	1195993..1197582	529		lysogenic conversion protein	Pseudomonas putida BIRD-1	YP_005932322.1	7E-69	154	527
**PSF113_0999**	CDS	-	1201927..1202331	134		hypothetical protein	Pseudomonas fluorescens Q8r1-96	ZP_17669922.1	1E-85	124	133
**PSF113_1038**	CDS	+	1240087..1240362	91		hypothetical protein	Pseudomonas syringae pv. aesculi str. NCPPB 3681	ZP_06459090.1	2E-54	80	91
**PSF113_1170**	CDS	+	1390188..1390769	193		dockerin type 1 protein	Pseudomonas chlororaphis O6	ZP_10174710.1	2E-107	158	193

F113 Locus	Type	Strand	Coordinates	Length (aa)	Gene	Function	Organism	Hit ACC	E-Value	Similarity	Alignment length
**PSF113_1178**	CDS	+	1396691..1397128	145		prophage tail fimber assembly protein	Pseudomonas chlororaphis subsp. aureofaciens 30-84	EJL08081.1	8E-09	25	37
**PSF113_1188**	CDS	-	1403843..1404490	215	rfaZ	RfaZ	Pseudomonas avellanae BPIC 631	ZP_16386226.1	2E-95	135	215
**PSF113_1463**	CDS	+	1686804..1687034	76		hypothetical protein	Pseudomonas sp. GM79	ZP_10612532.1	2E-41	63	76
**PSF113_1539**	CDS	+	1796727..1797500	257		Glucose-1-phosphate cytidylyltransferase (EC 2.7.7.33)	Pseudomonas sp. GM60	ZP_10641967.1	0	245	257
**PSF113_1540**	CDS	+	1797482..1798564	360		Similar to CDP-glucose 4,6-dehydratase (EC 4.2.1.45)	Pseudomonas sp. GM74	ZP_10629261.1	0	318	360
**PSF113_1541**	CDS	+	1798561..1799121	186		dTDP-4-dehydrorhamnose 3,5-epimerase	Pseudomonas sp. GM80	ZP_10606112.1	1E-123	170	186
**PSF113_1542**	CDS	+	1799121..1799990	289		UDP-glucose 4-epimerase	Pseudomonas sp. GM80	ZP_10606111.1	5E-169	233	284
**PSF113_1543**	CDS	+	1799980..1801050	356		C-methyltransferase	Pseudomonas sp. GM74	ZP_10629265.1	0	291	356
**PSF113_1544**	CDS	+	1801066..1801803	245		Cephalosporin hydroxylase	Pseudomonas fluorescens R124	ZP_18345414.1	5E-128	170	239
**PSF113_1547**	CDS	+	1805812..1806972	386		DegT/DnrJ/EryC1/StrS aminotransferase	Pseudomonas sp. GM17	ZP_10708182.1	0	320	386
**PSF113_1548**	CDS	+	1806979..1807680	233		N-Acetylneuraminate cytidylyltransferase	Pseudomonas sp. GM50	ZP_10649148.1	7E-141	199	231
**PSF113_1549**	CDS	+	1807677..1809182	501		Pseudaminic acid cytidylyltransferase (EC 2.7.7.43)	Pseudomonas fluorescens R124	ZP_18345418.1	0	379	501
**PSF113_1550**	CDS	+	1809175..1810227	350		N-acetylneuraminate synthase (EC 2.5.1.56)	Pseudomonas sp. GM80	ZP_10606103.1	0	312	350
**PSF113_1552**	CDS	+	1811395..1812726	443		Conserved domain protein	Pseudomonas sp. GM25	ZP_10688563.1	0	288	430
**PSF113_1553**	CDS	+	1812723..1812992	89		hypothetical protein	Pseudomonas sp. GM102	ZP_10594708.1	1E-25	49	84
**PSF113_1581**	CDS	-	1838167..1840368	733		hypothetical protein	Pseudomonas viridiflava UASWS0038	ZP_11289897.1	2E-92	167	369
**PSF113_1647**	CDS	+	1913982..1915241	419	matE	MatE	Pseudomonas aeruginosa UCBPP-PA14	YP_790024.1	2E-148	234	412
**PSF113_1651**	CDS	+	1918441..1919535	364		glycosyl hydrolase bnr repeat-containing protein	gamma proteobacterium HTCC2207	ZP_01225182.1	2E-80	132	342
**PSF113_1652**	CDS	+	1919557..1921701	714		putatve zinc-binding dehydrogenase	Ralstonia solanacearum CFBP2957	YP_003748271.1	0	487	686
**PSF113_1653**	CDS	+	1921710..1923356	548		Heparinase II/III-like	Pseudomonas aeruginosa UCBPP-PA14	YP_790028.1	0	360	547
**PSF113_1654**	CDS	+	1923359..1924543	394		hypothetical protein	Pseudomonas sp. GM67	ZP_10634475.1	0	257	388
**PSF113_1657**	CDS	+	1926872..1928065	397		acyltransferase 3	Pseudomonas sp. GM55	ZP_10643050.1	3E-160	235	398
**PSF113_1759**	CDS	-	2049894..2050790	298		Transcriptional regulator, LysR family	Pseudomonas sp. GM60	ZP_10636446.1	2E-171	242	295
**PSF113_1760**	CDS	+	2050894..2051742	282		ThiJ/PfpI family protein	Pseudomonas sp. GM67	ZP_10634853.1	3E-142	200	279
**PSF113_1793**	CDS	+	2090217..2090471	84	iacP	IacP	Pseudomonas sp. GM49	ZP_10654481.1	0,000001	21	37
**PSF113_1800**	CDS	+	2095052..2095618	188	orgA	OrgA	Pseudomonas fluorescens Q2-87	ZP_17084184.1	5E-89	138	182
**PSF113_1925**	CDS	-	2257555..2258784	409		Mobile element protein	Pseudomonas sp. GM79	ZP_10618130.1	0	280	349
**PSF113_1937**	CDS	+	2271783..2272937	384		hypothetical protein	Burkholderia phymatum STM815	YP_001863389.1	7E-36	108	307
**PSF113_2025**	CDS	-	2368301..2369224	307		Inositol transport system sugar-binding protein	Pseudomonas fluorescens SS101	ZP_17665903.1	0	259	307
**PSF113_2026**	CDS	-	2369273..2370427	384		Protein involved in biosynthesis of mitomycin antibiotics/polyketide fumonisin	Pseudomonas fragi A22	ZP_10850286.1	0	274	382
**PSF113_2027**	CDS	-	2370424..2371272	282		Dehydrogenases with different specificities (related to short-chain alcohol dehydrogenases)	Pseudomonas sp. GM78	ZP_10624378.1	0	271	282
**PSF113_2028**	CDS	+	2371397..2372422	341		dna-binding protein	Pseudomonas sp. M47T1	ZP_10148580.1	0	310	341
**PSF113_2029**	CDS	-	2372432..2373499	355	pnpD2	Alcohol dehydrogenase (EC 1.1.1.1)	Pseudomonas sp. GM78	ZP_10623894.1	0	333	354
**PSF113_2030**	CDS	-	2373504..2373917	137		hypothetical protein	Pseudomonas sp. GM79	ZP_10616321.1	8E-91	127	137
**PSF113_2031**	CDS	-	2373956..2374801	281	pcpA	PcpA	Pseudomonas sp. GM79	ZP_10616322.1	0	261	281
**PSF113_2032**	CDS	+	2374972..2375886	304		transcriptional regulator, LysR family	Pseudomonas sp. GM79	ZP_10616323.1	0	303	304
**PSF113_2034**	CDS	+	2377611..2379047	478		Permeases of the major facilitator superfamily	Pantoea sp. GM01	ZP_10556988.1	0	267	475
**PSF113_2035**	CDS	+	2379125..2380021	298		Catechol 1,2-dioxygenase 1 (EC 1.13.11.1)	Pseudomonas sp. GM79	ZP_10616326.1	0	291	298
**PSF113_2036**	CDS	+	2380211..2380945	244		Xylose isomerase domain protein TIM barrel	Pseudomonas sp. GM79	ZP_10616327.1	0	288	290
**PSF113_2037**	CDS	+	2381014..2382009	331		Myo-inositol 2-dehydrogenase (EC 1.1.1.18)	Pseudomonas sp. GM78	ZP_10623887.1	0	313	331
**PSF113_2038**	CDS	+	2382059..2383150	363		NADH-dependent dehydrogenase	Pseudomonas sp. GM78	ZP_10623886.1	0	356	363

F113 Locus	Type	Strand	Coordinates	Length (aa)	Gene	Function	Organism	Hit ACC	E-Value	Similarity	Alignment length
**PSF113_2042**	CDS	+	2387126..2387428	100		stress responsive alpha-beta barrel domain protein	Burkholderia sp. H160	ZP_03269771.1	3E-36	60	97
**PSF113_2145**	CDS	+	2513232..2513567	111		acetyltransferase, GNAT family	Pseudomonas mandelii JR-1	ZP_11108873.1	7E-55	83	107
**PSF113_2225**	CDS	-	2599711..2600664	317		endonuclease/exonuclease/phosphatase family	Pseudomonas aeruginosa PA7	YP_001348249.1	8E-35	103	312
**PSF113_2260**	CDS	+	2638804..2639544	246	azlC3	AzlC	Pseudomonas sp. GM60	ZP_10639841.1	6E-134	189	213
**PSF113_2261**	CDS	+	2639541..2639867	108		branched-chain amino acid transport	Pseudomonas sp. GM67	ZP_10632735.1	2E-54	99	108
**PSF113_2274**	CDS	+	2657055..2658269	404		Response regulator receiver domain protein	Nitrosospira multiformis ATCC 25196	YP_411242.1	6E-131	201	401
**PSF113_2442**	CDS	-	2850226..2851224	332		perosamine synthase	Pseudomonas sp. GM17	ZP_10709575.1	1E-154	229	293
**PSF113_2443**	CDS	-	2851215..2851724	169		hypothetical protein	Pseudomonas sp. GM17	ZP_10709576.1	7E-107	155	169
**PSF113_2445**	CDS	-	2862223..2863581	452		hypothetical protein	Nitrolancetus hollandicus Lb	ZP_10246177.1	2E-91	204	472
**PSF113_2447**	CDS	-	2867162..2867848	228		hypothetical protein	Pseudomonas fragi A22	ZP_10849905.1	12	38	123
**PSF113_2448**	CDS	-	2868202..2868537	111		hypothetical protein	Nitrolancetus hollandicus Lb	ZP_10246176.1	2E-09	37	107
**PSF113_2449**	CDS	-	2868865..2869620	251		iron ABC transporter ATP-binding protein	Pseudomonas sp. GM67	ZP_10632022.1	1E-164	231	251
**PSF113_2450**	CDS	-	2869614..2870675	353	fecD	Iron(III) dicitrate transport system permease protein FecD (TC 3.A.1.14.1)	Pseudomonas mandelii JR-1	ZP_11114318.1	0	325	352
**PSF113_2451**	CDS	-	2870672..2871859	395		periplasmic binding protein	Pseudomonas sp. GM49	ZP_10655015.1	0	339	395
**PSF113_2453**	CDS	-	2874435..2875412	325		Fe2+-dicitrate sensor,membrane component	Pseudomonas sp. GM41(2012)	ZP_10670704.1	0	278	325
**PSF113_2454**	CDS	-	2875413..2875946	177		RNA polymerase sigma-70 factor, ECF subfamily	Pseudomonas sp. GM41(2012)	ZP_10670703.1	6E-112	157	171
**PSF113_2455**	CDS	-	2876015..2877241	408		Multidrug resistance protein B	Pseudomonas sp. GM67	ZP_10632028.1	0	331	403
**PSF113_2468**	CDS	-	2889865..2895588	1907		hypothetical protein	Pseudomonas sp. GM41(2012)	ZP_10667339.1	3E-23	187	654
**PSF113_2571**	CDS	-	3027075..3028190	371		Phenylpropionate dioxygenase and related ring-hydroxylating dioxygenases, large terminal subunit	Pseudomonas aeruginosa ATCC 700888	ZP_15626210.1	0	273	364
**PSF113_2574**	CDS	-	3030979..3031806	275		transcriptional regulatory protein	Burkholderia sp. Ch1-1	ZP_10031946.1	2E-41	85	267
**PSF113_2578**	CDS	-	3035847..3036497	216		hypothetical protein	Leptosphaeria maculans JN3	XP_003840512.1	2E-30	79	225
**PSF113_2580**	CDS	-	3039138..3040232	364		bnr domain-containing protein	Pseudomonas fluorescens Q8r1-96	ZP_17671118.1	4E-91	170	358
**PSF113_2583**	CDS	+	3043484..3044029	181		hypothetical protein	Sphingobium sp. SYK-6	YP_004834889.1	2E-45	76	160
**PSF113_2584**	CDS	+	3044049..3044504	151		aromatic-ring-hydroxylating dioxygenase beta subunit	Sphingobium sp. SYK-6	YP_004835931.1	6E-27	59	147
**PSF113_2585**	CDS	+	3044563..3045504	313		NmrA-like protein	Frankia sp. QA3	ZP_10309179.1	3E-105	172	299
**PSF113_2605**	CDS	-	3065028..3065993	321	pdtC	PdtC, QsbA	Pseudomonas sp. GM102	ZP_10599892.1	0	287	321
**PSF113_2607**	CDS	+	3068150..3069193	347	ptdP	PtdP, QbsJ	Pseudomonas sp. GM102	ZP_10599890.1	0	278	343
**PSF113_2608**	CDS	+	3069190..3070482	430	ampG2	AmpG	Pseudomonas sp. GM50	ZP_10650419.1	2E-174	309	407
**PSF113_2609**	CDS	+	3070641..3072908	755		Methionine ABC transporter ATP-binding protein	Ralstonia solanacearum GMI1000	NP_521762.1	0	537	808
**PSF113_2610**	CDS	+	3072932..3075454	840		Putrescine aminotransferase (EC 2.6.1.82)	Serratia sp. AS12	YP_004500188.1	0	433	852
**PSF113_2611**	CDS	+	3075828..3076871	347	pdtF	Sulfur carrier protein adenylyltransferase ThiF	Pseudomonas sp. GM102	ZP_10599886.1	0	308	347
**PSF113_2612**	CDS	+	3076893..3077300	135	pdtG	PdtG, QsbD	Pseudomonas sp. GM50	ZP_10650423.1	1E-79	117	134
**PSF113_2613**	CDS	+	3077372..3077644	90	pdtH	PdtH, QsbE	Pseudomonas sp. GM102	ZP_10599884.1	7E-50	78	90
**PSF113_2614**	CDS	+	3077729..3079582	617		hypothetical protein	Pseudomonas sp. TJI-51	ZP_08143500.1	0	298	547
**PSF113_2615**	CDS	+	3079579..3081228	549	pdtJ	Long-chain-fatty-acid--CoA ligase (EC 6.2.1.3)	Ralstonia solanacearum Po82	YP_006032419.1	0	378	548
**PSF113_2616**	CDS	+	3081267..3082442	391	pdtO	PdtO	Pseudomonas sp. GM50	ZP_10650427.1	0	344	391
**PSF113_2617**	CDS	+	3082439..3083644	401		hypothetical protein	Pseudomonas sp. GM50	ZP_10650428.1	0	334	402
**PSF113_2618**	CDS	+	3083641..3084723	360		unsaturated glucuronyl hydrolase	Pseudomonas sp. GM102	ZP_10599879.1	2E-150	242	366
**PSF113_2736**	CDS	+	3218181..3219521	446	tolC	TolC	Pseudomonas mendocina ymp	YP_001186218.1	7E-167	258	432
**PSF113_2750**	CDS	+	3234170..3234487	105		transcriptional regulator	Pseudomonas sp. GM74	ZP_10629848.1	2E-64	100	105
**PSF113_2751**	CDS	+	3234480..3235751	423	hipA2	HIPA PROTEIN	Pseudomonas fluorescens WH6	ZP_07776376.1	0	398	419
**PSF113_2752**	CDS	+	3236163..3237206	347		Dihydroflavonol-4-reductase (EC 1.1.1.219)	Pseudomonas sp. HYS	ZP_11261341.1	0	254	347
**PSF113_2865**	CDS	+	3375210..3375602	130		putative translation initiation inhibitor protein	Novosphingobium sp. AP12	ZP_10744851.1	7E-82	116	130
**PSF113_2866**	CDS	+	3375665..3376489	274		Saccharopine dehydrogenase	Pseudomonas sp. GM24	ZP_10694342.1	4E-87	124	168

F113 Locus	Type	Strand	Coordinates	Length (aa)	Gene	Function	Organism	Hit ACC	E-Value	Similarity	Alignment length
**PSF113_2875**	CDS	+	3385596..3386573	325		probable transcription regulator PA3771	Pseudomonas chlororaphis O6	ZP_10175621.1	0	269	325
**PSF113_2876**	CDS	+	3386711..3387586	291	qbdB	QbdB	Pseudomonas sp. GM17	ZP_10712333.1	0	263	291
**PSF113_2877**	CDS	+	3387609..3388772	387		Permeases of the major facilitator superfamily	Pseudomonas aeruginosa 138244	ZP_11915370.1	9E-139	261	351
**PSF113_2878**	CDS	+	3388807..3389919	370		Deacetylases, including yeast histone deacetylase and acetoin utilization protein	Pseudomonas aeruginosa PAb1	ZP_06877188.1	0	270	365
**PSF113_3032**	CDS	-	3583921..3584643	240		hypothetical protein	Pseudomonas mandelii JR-1	ZP_11110187.1	8E-151	208	239
**PSF113_3033**	CDS	-	3584806..3585750	314		hypothetical protein	Pseudomonas viridiflava UASWS0038	ZP_11286954.1	2E-87	159	317
**PSF113_3034**	CDS	-	3585747..3587411	554		hypothetical protein	Pseudomonas viridiflava UASWS0038	ZP_11286953.1	0	302	566
**PSF113_3036**	CDS	-	3589239..3589994	251		cobalamin biosynthetic protein	Pseudomonas protegens Pf-5	YP_259560.1	8E-93	139	239
**PSF113_3123**	CDS	-	3682946..3684298	450		serine protease,subtilase family	Arsenophonus nasoniae	CBA74876.1	1E-108	204	430
**PSF113_3132**	CDS	-	3692953..3694023	356		putative exported protein	Rhizobium leguminosarum bv. trifolii WSM1325	YP_002976281.1	0	244	329
**PSF113_3136**	CDS	-	3696580..3697176	198		twin-arginine translocation pathway signal	Pseudomonas sp. GM24	ZP_10691545.1	7E-129	178	194
**PSF113_3151**	CDS	-	3712292..3714682	796		Ferrichrome-iron receptor	Halomonas boliviensis LC1	ZP_09186870.1	0	464	661
**PSF113_3153**	CDS	-	3715768..3716166	132		RNA polymerase sigma-70 factor, ECF subfamily	Pseudomonas sp. GM80	ZP_10608172.1	5E-85	128	132
**PSF113_3157**	CDS	-	3717668..3718996	442		periplasmic serine protease	Pseudomonas sp. GM67	ZP_10632736.1	5E-28	64	151
**PSF113_3298**	CDS	-	3889413..3889667	84		putative membrane protein	Pseudomonas sp. GM102	ZP_10597742.1	2E-44	75	84
**PSF113_3302**	CDS	+	3892671..3892844	57		hypothetical protein	Pseudomonas fluorescens Pf0-1	YP_348753.1	9E-28	51	53
**PSF113_3303**	CDS	+	3893476..3894552	358		putative membrane protein	Pseudomonas stutzeri DSM 10701	YP_006522558.1	5E-171	254	330
**PSF113_3305**	CDS	+	3897288..3898403	371		ABC-type multidrug transport system, permease component	Pseudomonas stutzeri A1501	YP_001170635.1	0	273	371
**PSF113_3306**	CDS	-	3898507..3898764	85		Acetate kinase	Rhodanobacter sp. 2APBS1	ZP_08953387.1	2E-16	44	78
**PSF113_3308**	CDS	+	3900162..3902099	645		hypothetical protein	Xanthomonas campestris pv. campestris str. ATCC 33913	NP_636148.1	0	321	642
**PSF113_3309**	CDS	+	3902096..3903097	333		putative membrane protein	Pseudomonas stutzeri TS44	ZP_14470735.1	3E-143	202	322
**PSF113_3310**	CDS	+	3903240..3904277	345		Threonine dehydrogenase and related Zn-dependent dehydrogenases	Celeribacter baekdonensis B30	ZP_11133190.1	0	251	344
**PSF113_3311**	CDS	+	3904883..3906370	495		Membrane protein	Pseudomonas sp. Ag1	ZP_10475632.1	0	384	455
**PSF113_3313**	CDS	-	3906857..3908554	565		Sulfate permease	Pseudomonas entomophila L48	YP_609791.1	0	464	561
**PSF113_3350**	CDS	-	3943406..3944590	394		3-ketoacyl-CoA thiolase(EC 2.3.1.16) @ Acetyl-CoA acetyltransferase (EC 2.3.1.9)	Pseudomonas sp. HYS	ZP_11258811.1	0	315	394
**PSF113_3352**	CDS	+	3945924..3947576	550		Histidine kinase, HAMP region:Bacterial chemotaxis sensory transducer precursor	Pseudomonas stutzeri TS44	ZP_14470700.1	0	372	549
**PSF113_3353**	CDS	-	3947629..3947958	109		putative MarR family transcriptional regulator	Pseudomonas sp. GM21	ZP_10695694.1	7E-57	87	108
**PSF113_3355**	CDS	-	3948810..3952373	1187		Indolepyruvate ferredoxin oxidoreductase, alpha and beta subunits	Chromobacterium violaceum ATCC 12472	NP_899973.1	0	694	1138
**PSF113_3356**	CDS	+	3952747..3954417	556		Anaerobic dehydrogenases,typically selenocysteine-containing	Pseudomonas stutzeri TS44	ZP_14470659.1	0	413	547
**PSF113_3363**	CDS	+	3960840..3961766	308		Quinone oxidoreductase(EC 1.6.5.5)	Pseudomonas sp. GM21	ZP_10695681.1	0	292	308
**PSF113_3365**	CDS	+	3962878..3963765	295	cysB3	Cys regulon transcriptional activator CysB	Pseudomonas sp. GM21	ZP_10695680.1	0	264	295
**PSF113_3366**	CDS	+	3963931..3964968	345	ditL	2-amino-3-carboxymuconate-6-semialdehyde decarboxylase (EC 4.1.1.45)	Pseudomonas aeruginosa 2192	ZP_04934604.1	0	279	337
**PSF113_3367**	CDS	-	3965013..3965858	281		3-oxoacyl-[acyl-carrier protein] reductase (EC 1.1.1.100)	Pseudomonas sp. GM21	ZP_10695668.1	0	268	281
**PSF113_3368**	CDS	+	3966280..3967941	553		putative exported protein	Pseudomonas aeruginosa 2192	ZP_04934658.1	0	368	555
**PSF113_3370**	CDS	+	3969429..3970559	376		glycosyl hydrolase	Pseudomonas sp. GM21	ZP_10695665.1	0	319	376
**PSF113_3372**	CDS	+	3973174..3974145	323		Lactoylglutathione lyase-related lyase	Glaciecola sp. 4H-3-7+YE-5	YP_004432635.1	0	247	320
**PSF113_3373**	CDS	+	3974142..3974960	272		Enoyl-CoA hydratase (EC 4.2.1.17)	Pseudomonas sp. GM21	ZP_10695662.1	6E-168	253	272
**PSF113_3375**	CDS	+	3975905..3977509	534		Beta-carotene ketolase(EC 1.14.-.-)	Marinobacter adhaerens HP15	YP_005883709.1	0	420	529

F113 Locus	Type	Strand	Coordinates	Length (aa)	Gene	Function	Organism	Hit ACC	E-Value	Similarity	Alignment length
**PSF113_3376**	CDS	-	3977567..3978232	221		transcriptional regulator, TetR family	Pseudomonas sp. GM21	ZP_10695659.1	7E-150	208	221
**PSF113_3377**	CDS	+	3978415..3979692	425	ditQ	Cytochrome P450	Marinobacter adhaerens HP15	YP_005883710.1	0	273	418
**PSF113_3378**	CDS	+	3979743..3980774	343		hypothetical protein	Pseudomonas mendocina DLHK	ZP_15951229.1	0	302	343
**PSF113_3380**	CDS	+	3981696..3982997	433		Permeases of the major facilitator superfamily	Pseudomonas sp. GM21	ZP_10695655.1	0	382	433
**PSF113_3381**	CDS	+	3983021..3983371	116		hypothetical protein	Pseudomonas sp. GM21	ZP_10695654.1	3E-74	106	116
**PSF113_3382**	CDS	-	3983428..3984261	277		short-chain dehydrogenase reductase sdr	Herbaspirillum sp. CF444	ZP_10722646.1	9E-87	136	275
**PSF113_3383**	CDS	-	3984344..3985579	411	ethA	Ferredoxin reductase	Pseudomonas stutzeri TS44	ZP_14470697.1	0	341	411
**PSF113_3384**	CDS	-	3985600..3985920	106		Ferredoxin, 2Fe-2S	Pseudomonas sp. GM21	ZP_10695651.1	2E-68	99	106
**PSF113_3385**	CDS	-	3985954..3987216	420		major facilitator superfamily mfs_1	Pseudomonas aeruginosa 2192	ZP_04934635.1	0	323	398
**PSF113_3386**	CDS	+	3987569..3987805	78	ditA3	DitA3	Pseudomonas abietaniphila	AAD21062.1	2E-37	60	77
**PSF113_3387**	CDS	+	3987851..3988615	254	ditB	Enoyl-[acyl-carrier-protein] reductase [NADH] (EC 1.3.1.9)	Pseudomonas sp. GM21	ZP_10695648.1	2E-173	242	252
**PSF113_3388**	CDS	+	3988677..3989627	316	ditC	2,3-dihydroxybiphenyl 1,2-dioxygenase (EC 1.13.11.39)	Pseudomonas aeruginosa 2192	ZP_04934631.1	0	263	301
**PSF113_3389**	CDS	+	3989629..3990513	294	ditD	Fumarylacetoacetate hydrolase family protein	Pseudomonas sp. GM21	ZP_10695646.1	0	259	294
**PSF113_3390**	CDS	+	3990548..3990832	94	ditE	DitE	Pseudomonas sp. GM21	ZP_10695645.1	5E-42	73	83
**PSF113_3391**	CDS	+	3990897..3991682	261	ditR	Transcriptional regulator, IclR family	Bordetella bronchiseptica Bbr77	CCN03081.1	7E-43	85	235
**PSF113_3392**	CDS	-	3991692..3992882	396	ditF	3-ketoacyl-CoA thiolase(EC 2.3.1.16)	Marinobacter adhaerens HP15	YP_005883727.1	0	305	382
**PSF113_3393**	CDS	-	3992882..3993280	132		hypothetical protein	Pseudomonas sp. GM21	ZP_10695642.1	5E-87	124	132
**PSF113_3394**	CDS	+	3993420..3994121	233	ditG	DitG	Pseudomonas sp. GM21	ZP_10695641.1	6E-150	217	233
**PSF113_3395**	CDS	+	3994127..3995116	329	ditH	Fumarylacetoacetate hydrolase family protein	Pseudomonas mendocina DLHK	ZP_15951247.1	0	276	325
**PSF113_3396**	CDS	+	3995231..3996631	466	ditA1	Large subunit naph/bph dioxygenase	Pseudomonas aeruginosa 2192	ZP_04934639.1	0	318	465
**PSF113_3397**	CDS	+	3996721..3997296	191	ditA2	Biphenyl dioxygenase beta subunit (EC 1.14.12.18)	Pseudomonas sp. GM21	ZP_10695638.1	2E-127	181	191
**PSF113_3398**	CDS	-	3997382..3998122	246		hypothetical protein	Pseudomonas sp. GM21	ZP_10695637.1	2E-134	208	244
**PSF113_3399**	CDS	+	3998224..3999024	266	ditI	Putative short-chain dehydrogenase/reductase (EC 1.1.1.100)	Pseudomonas sp. GM21	ZP_10695636.1	0	261	266
**PSF113_3400**	CDS	-	3999151..4000782	543	ditJ	Long-chain-fatty-acid--CoA ligase (EC 6.2.1.3)	Frankia sp. EUN1f	ZP_06413672.1	2E-177	257	528
**PSF113_3401**	CDS	+	4000967..4001698	243	ditK	DitK	Pseudomonas sp. GM21	ZP_10695634.1	4E-137	192	196
**PSF113_3402**	CDS	+	4002263..4003096	277	ditL2	2-amino-3-carboxymuconate-6-semialdehyde decarboxylase (EC 4.1.1.45)	Pseudomonas sp. GM21	ZP_10695633.1	0	265	277
**PSF113_3403**	CDS	+	4003096..4003947	283	ditM	Fumarylacetoacetate hydrolase family protein	Pseudomonas sp. GM21	ZP_10695632.1	0	269	283
**PSF113_3404**	CDS	+	4003961..4004863	300	ditN	3-hydroxybutyryl-CoA dehydrogenase (EC 1.1.1.157); 3-hydroxyacyl-CoA dehydrogenase (EC 1.1.1.35)	Pseudomonas sp. GM21	ZP_10695631.1	0	289	300
**PSF113_3405**	CDS	+	4004860..4006035	391	ditO	3-ketoacyl-CoA thiolase(EC 2.3.1.16) @ Acetyl-CoA acetyltransferase (EC 2.3.1.9)	Burkholderia xenovorans LB400	YP_555845.1	0	283	389
**PSF113_3407**	CDS	+	4006680..4007957	425	ditQ2	Cytochrome P450	Glaciecola sp. 4H-3-7+YE-5	YP_004432599.1	0	302	424
**PSF113_3408**	CDS	+	4008071..4009378	435		BarH	Glaciecola sp. 4H-3-7+YE-5	YP_004432600.1	0	345	435
**PSF113_3409**	CDS	+	4009615..4010637	340		FadE30	Pseudomonas mendocina DLHK	ZP_15951261.1	0	280	322
**PSF113_3410**	CDS	+	4010637..4011710	357		Acyl-CoA dehydrogenase,short-chain specific	Pseudomonas sp. GM21	ZP_10696717.1	0	319	357
**PSF113_3411**	CDS	+	4011707..4012507	266		Enoyl-CoA hydratase (EC 4.2.1.17)	Pseudomonas sp. GM21	ZP_10696716.1	0	258	266
**PSF113_3412**	CDS	+	4012550..4013674	374		Acyl-CoA dehydrogenase,short-chain specific (EC 1.3.99.2)	Pseudomonas aeruginosa 2192	ZP_04934607.1	0	344	374
**PSF113_3413**	CDS	+	4013699..4014250	183		Glutathione peroxidase(EC 1.11.1.9)	Pseudomonas sp. GM21	ZP_10696714.1	6E-115	159	183
**PSF113_3414**	CDS	+	4014253..4015113	286		Pantoate--beta-alanine ligase (EC 6.3.2.1)	Pseudomonas sp. GM21	ZP_10696713.1	2E-168	231	286
**PSF113_3415**	CDS	+	4015202..4016059	285		methyl-accepting chemotaxis protein	Pseudomonas sp. GM21	ZP_10696712.1	0	256	285
**PSF113_3416**	CDS	+	4016066..4017271	401		major facilitator superfamily transporter	Pseudomonas sp. GM21	ZP_10696710.1	0	362	401
**PSF113_3439**	CDS	+	4050097..4051026	309	pnpR	Transcriptional regulator, LysR family	Pseudomonas sp. NyZ402	ACZ51387.1	0	263	308
**PSF113_3440**	CDS	+	4051143..4051637	164	pnpC1	PnpC1	Pseudomonas sp. GM78	ZP_10624263.1	4E-107	149	164

F113 Locus	Type	Strand	Coordinates	Length (aa)	Gene	Function	Organism	Hit ACC	E-Value	Similarity	Alignment length
**PSF113_3441**	CDS	+	4051691..4052710	339	pnpC2	FIG00454641: hypothetical protein	Burkholderia sp. CCGE1002	YP_003609571.1	0	271	339
**PSF113_3442**	CDS	+	4052805..4054268	487	pnpD	Aldehyde dehydrogenase(EC 1.2.1.3)	Burkholderia ambifaria AMMD	YP_777096.1	0	394	488
**PSF113_3443**	CDS	+	4054277..4055344	355	pnpF	Alcohol dehydrogenase (EC 1.1.1.1)	Pseudomonas sp. WBC-3	ABU50914.1	0	327	355
**PSF113_3444**	CDS	+	4055420..4056292	290	dio1	Dio1	Pseudomonas sp. WBC-3	ABU50913.1	0	247	290
**PSF113_3445**	CDS	+	4056296..4056607	103	yciL	YciL	Pseudomonas sp. GM78	ZP_10624268.1	1E-60	90	103
**PSF113_3446**	CDS	+	4056609..4056956	115	pnpX2	PnpX2	Pseudomonas sp. GM78	ZP_10624269.1	2E-63	91	111
**PSF113_3450**	CDS	-	4060077..4060928	283	hpcE	HpcE	Bradyrhizobium sp. WSM471	ZP_09649218.1	5E-95	150	281
**PSF113_3451**	CDS	+	4061690..4062052	120		4-hydroxyphenylacetate 3-monooxygenase reductase component	Granulibacter bethesdensis CGDNIH1	YP_744202.1	1E-33	58	112
**PSF113_3452**	CDS	-	4062156..4062659	167		carboxymuconolactone decarboxylase	Alicycliphilus denitrificans BC	YP_004128766.1	2E-65	107	165
**PSF113_3456**	CDS	+	4066299..4067786	495		3-(3-hydroxy-phenyl)propionate hydroxylase	Agrobacterium vitis S4	YP_002539780.1	0	314	488
**PSF113_3457**	CDS	+	4067890..4068525	211		arylmalonate decarboxylase	Novosphingobium sp. Rr 2-17	ZP_10362077.1	2E-70	114	207
**PSF113_3459**	CDS	+	4070563..4070994	143		putative exported protein	Agrobacterium vitis S4	YP_002539772.1	2E-44	75	139
**PSF113_3460**	CDS	-	4071116..4071901	261		mhp operon transcriptional activator	Agrobacterium vitis S4	YP_002539763.1	3E-66	107	253
**PSF113_3463**	CDS	+	4074715..4075464	249		carboxymuconolactone decarboxylase	Agrobacterium vitis S4	YP_002539775.1	1E-71	114	213
**PSF113_3466**	CDS	+	4077150..4077575	141		carboxymuconolactone decarboxylase	Agrobacterium vitis S4	YP_002539778.1	1E-55	88	137
**PSF113_3467**	CDS	+	4077610..4078158	182		Biphenyl-2,3-diol 1,2-dioxygenase	Novosphingobium sp. Rr 2-17	ZP_10362079.1	6E-56	86	171
**PSF113_3469**	CDS	+	4079513..4079845	110		nucleic-acid-binding protein containing a zn-ribbon	Novosphingobium sp. Rr 2-17	ZP_10362075.1	1E-27	50	80
**PSF113_3476**	CDS	-	4087478..4088362	294	ligR	Transcriptional regulator ligR, LysR family	Pseudomonas putida W619	YP_001748333.1	2E-141	201	288
**PSF113_3477**	CDS	-	4088390..4089316	308		3-hydroxyisobutyrate dehydrogenase-related beta-hydroxyacid dehydrogenase	Pseudomonas sp. M47T1	ZP_10149287.1	1E-113	192	282
**PSF113_3479**	CDS	-	4090410..4091126	238		Putative siderophore biosynthesis protein, related to 2-demethylmenaquinone methyltransferase	Serratia proteamaculans 568	YP_001478322.1	5E-127	177	235
**PSF113_3480**	CDS	-	4091123..4091857	244		FIG00959802: hypothetical protein	Pseudomonas syringae pv. aesculi str. NCPPB 3681	ZP_06460101.1	8E-168	223	244
**PSF113_3481**	CDS	+	4092011..4093234	407		Transcriptional regulator, LysR family	Pseudomonas sp. GM78	ZP_10623222.1	0	270	403
**PSF113_3483**	CDS	+	4094091..4095257	388		Lysophospholipase (EC 3.1.1.5)	Desulfurispirillum indicum S5	YP_004113620.1	3E-171	233	379
**PSF113_3497**	CDS	-	4109894..4111024	376		Multidrug resistance efflux pump	Agrobacterium sp. H13-3	YP_004280648.1	6E-143	216	363
**PSF113_3498**	CDS	-	4111098..4111544	148		putative membrane protein	Pseudomonas sp. GM18	ZP_10704146.1	2E-69	106	144
**PSF113_3563**	CDS	+	4185660..4186736	358	cheB3	Chemotaxis response regulator protein-glutamate methylesterase CheB (EC 3.1.1.61)	Pseudomonas extremaustralis 14-3 substr. 14-3b	ZP_10435492.1	0	287	354
**PSF113_3573**	CDS	-	4200744..4201667	307		Transcriptional regulator, LysR family	Pseudomonas sp. GM79	ZP_10614327.1	1E-145	206	300
**PSF113_3574**	CDS	-	4201701..4202675	324		Quinone oxidoreductase(EC 1.6.5.5)	Pseudomonas syringae pv. aceris str. M302273	ZP_16733109.1	3E-172	244	324
**PSF113_3582**	CDS	+	4212409..4213761	450		2-methylcitrate dehydratase	Alcanivorax dieselolei B5	YP_006821692.1	0	304	434
**PSF113_3583**	CDS	-	4213812..4215017	401	xis	Xis	Alcanivorax dieselolei B5	YP_006821700.1	1E-158	232	389
**PSF113_3584**	CDS	+	4215219..4216565	448		Citrate transporter	Pseudomonas mendocina NK-01	YP_004380609.1	0	264	423
**PSF113_3586**	CDS	+	4218078..4219106	342		D-3-phosphoglycerate dehydrogenase (EC 1.1.1.95)	Pseudomonas sp. Ag1	ZP_10477217.1	5E-139	211	332
**PSF113_3587**	CDS	+	4219103..4220470	455		3-methylaspartate ammonia-lyase, glutamate mutase	Pseudomonas sp. Ag1	ZP_10477218.1	0	320	452
**PSF113_3588**	CDS	+	4220472..4220798	108		hypothetical protein	Pseudomonas sp. Ag1	ZP_10477219.1	2E-36	56	100
**PSF113_3590**	CDS	+	4221775..4223043	422		secretory lipase	Alcanivorax dieselolei B5	YP_006821703.1	1E-180	274	403
**PSF113_3656**	CDS	-	4302243..4303679	478		drug resistance mfs transporter	Burkholderia multivorans ATCC BAA-247	ZP_15919178.1	4E-159	264	437
**PSF113_3660**	CDS	+	4315325..4316173	282	lkcD	LkcD	Hahella chejuensis KCTC 2396	YP_434659.1	1E-97	149	283
**PSF113_3663**	CDS	+	4318277..4325512	2411	lkcF	Malonyl CoA-acyl carrier protein transacylase (EC 2.3.1.39)	Hahella chejuensis KCTC 2396	YP_434656.1	2E-50	182	571
**PSF113_3666**	CDS	+	4329095..4330678	527	lkcJ	LkcJ	Pseudomonas sp. Ag1	ZP_10474276.1	0	433	525

F113 Locus	Type	Strand	Coordinates	Length (aa)	Gene	Function	Organism	Hit ACC	E-Value	Similarity	Alignment length
**PSF113_4073**	CDS	-	4801978..4802601	207		putative lipoprotein	Pseudomonas protegens Pf-5	YP_260543.1	1E-51	96	179
**PSF113_4191**	CDS	-	4918510..4918704	64		hypothetical protein	Pseudomonas sp. GM79	ZP_10614059.1	1E-32	58	64
**PSF113_4279**	CDS	+	5007641..5008678	345		DNA-cytosine methyltransferase (EC 2.1.1.37)	Pseudomonas syringae pv. tomato Max13	ZP_07234026.1	0	287	344
**PSF113_4283**	CDS	+	5013638..5014042	134		Gifsy-2 prophage protein	Pseudomonas fluorescens BBc6R8	ZP_15597875.1	2E-43	71	97
**PSF113_4290**	CDS	-	5018559..5021138	859		Methyl-accepting chemotaxis protein	Pseudomonas sp. GM41(2012)	ZP_10670376.1	0	351	534
**PSF113_4291**	CDS	-	5021154..5021276	40		hypothetical protein	Pseudomonas syringae pv. syringae FF5	ZP_06500139.1	1E-08	23	40
**PSF113_4292**	CDS	-	5021239..5021565	108		hypothetical protein	Pseudomonas entomophila L48	YP_609626.1	1E-38	62	94
**PSF113_4299**	CDS	-	5027777..5027926	49		hypothetical protein	Pseudomonas fluorescens WH6	ZP_07773798.1	4E-18	34	49
**PSF113_4300**	CDS	-	5028086..5028649	187		Baseplate assembly protein V	Pseudomonas syringae pv. tomato K40	ZP_07255094.1	1E-108	152	187
**PSF113_4301**	CDS	-	5028646..5029170	174		hypothetical protein	Pseudomonas syringae pv. lachrymans str. M302278	ZP_16721365.1	6E-74	123	170
**PSF113_4302**	CDS	-	5029163..5029825	220		FIG00959540: hypothetical protein	Pseudomonas sp. GM17	ZP_10710365.1	3E-119	165	215
**PSF113_4303**	CDS	-	5029822..5030136	104		hypothetical protein	Pseudomonas putida GB-1	YP_001667445.1	6E-49	82	104
**PSF113_4304**	CDS	-	5030139..5031134	331		elements of external origin; phage-related functions and prophages	Pseudomonas mandelii JR-1	ZP_11109272.1	0	292	331
**PSF113_4305**	CDS	-	5031478..5031822	114		hypothetical protein	Pseudomonas mandelii JR-1	ZP_11112469.1	2E-64	99	114
**PSF113_4306**	CDS	-	5031819..5032979	386		Prophage Clp protease-like protein	Pseudomonas sp. GM74	ZP_10626716.1	0	324	383
**PSF113_4307**	CDS	-	5032976..5034457	493		FIG00963359: hypothetical protein	Pseudomonas syringae pv. actinidiae str. M302091	ZP_16713621.1	0	386	493
**PSF113_4308**	CDS	-	5034457..5034663	68		hypothetical protein	Pseudomonas sp. GM41(2012)	ZP_10670358.1	7E-39	63	68
**PSF113_4309**	CDS	-	5034665..5036677	670		Phage terminase, large subunit	Pseudomonas fulva 12-X	YP_004473518.1	0	430	606
**PSF113_4310**	CDS	-	5036682..5037284	200		terminase small subunit	Pseudomonas sp. GM41(2012)	ZP_10670356.1	2E-123	174	200
**PSF113_4312**	CDS	-	5038288..5038647	119		hypothetical protein	Pseudomonas fluorescens WH6	ZP_07773787.1	2E-66	100	119
**PSF113_4313**	CDS	-	5038640..5040859	739		DNA primase, phage associated	Pseudomonas sp. GM80	ZP_10611520.1	0	623	739
**PSF113_4314**	CDS	-	5040849..5041076	75		C4-type zinc finger protein, DksA/TraR family	Pseudomonas syringae pv. lachrymans str. M302278	ZP_16721378.1	6E-33	58	75
**PSF113_4315**	CDS	-	5041069..5041587	172		FIG00958730: hypothetical protein	Pseudomonas syringae pv. lachrymans str. M302278	ZP_16721379.1	6E-109	153	172
**PSF113_4319**	CDS	+	5044275..5044859	194		hypothetical protein	Pseudomonas fluorescens BBc6R8	ZP_15597836.1	5E-93	136	185
**PSF113_4320**	CDS	+	5044870..5045154	94		DNA-binding protein Roi-related protein	Pseudomonas protegens Pf-5	YP_259102.1	8E-54	83	94
**PSF113_4321**	CDS	+	5045313..5045642	109		transcriptional regulator	Pseudomonas protegens Pf-5	YP_259101.1	1E-60	92	108
**PSF113_4322**	CDS	+	5045686..5046102	138		hypothetical protein	Pseudomonas sp. GM74	ZP_10626698.1	2E-44	77	130
**PSF113_4323**	CDS	+	5046099..5046320	73		hypothetical protein	Pseudomonas fluorescens WH6	ZP_07773778.1	7E-27	51	73
**PSF113_4324**	CDS	+	5046317..5046859	180		metal dependent phosphohydrolase	Pseudomonas sp. GM41(2012)	ZP_10670343.1	1E-104	148	180
**PSF113_4325**	CDS	+	5047281..5048489	402		phage integrase	Pseudomonas extremaustralis 14-3 substr. 14-3b	ZP_10438956.1	0	267	336
**PSF113_4501**	CDS	-	5257976..5259292	438		hipa-like c-terminal domain protein	Pseudomonas putida LS46	ZP_15971311.1	0	277	438
**PSF113_4845**	CDS	+	5633910..5634398	162		RNA polymerase sigma-70 factor, ECF subfamily	Pseudomonas sp. GM60	ZP_10637073.1	4E-96	138	162
**PSF113_4846**	CDS	+	5634407..5635354	315		Iron siderophore sensor protein	Pseudomonas sp. GM78	ZP_10618249.1	5E-153	215	312
**PSF113_4889**	CDS	+	5680747..5680911	54		hypothetical protein	Pseudomonas brassicacearum subsp. brassicacearum NFM421	YP_004356144.1	1E-29	50	54
**PSF113_4945**	CDS	+	5740706..5740900	64		hypothetical protein	Pseudomonas sp. R81	ZP_11190749.1	3E-27	50	64
**PSF113_4946**	CDS	+	5741299..5742384	361		filamentation induced by cAMP protein fic	Pseudomonas syringae pv. avellanae str. ISPaVe037	ZP_17811982.1	0	334	361

F113 Locus	Type	Strand	Coordinates	Length (aa)	Gene	Function	Organism	Hit ACC	E-Value	Similarity	Alignment length
**PSF113_5105**	CDS	-	5915647..5916246	199		Transcriptional regulator, GntR family	Pseudomonas syringae pv. morsprunorum str. M302280	ZP_16662817.1	1E-123	172	199
**PSF113_5106**	CDS	+	5916647..5917816	389		Mn2+/Fe2+ transporter,NRAMP family	Pseudomonas syringae pv. oryzae str. 1_6	ZP_04586656.1	0	306	389
**PSF113_5184**	CDS	+	6003200..6005176	658		Type I restriction-modification system, DNA-methyltransferase subunit M (EC 2.1.1.72)	Pseudomonas sp. GM17	ZP_10707280.1	0	594	661
**PSF113_5185**	CDS	+	6005173..6006546	457		Type I restriction-modification system, specificity subunit S (EC 3.1.21.3)	Pseudomonas aeruginosa ATCC 14886	ZP_15611258.1	2E-143	234	439
**PSF113_5186**	CDS	+	6006546..6009743	1065		Type I restriction-modification system, restriction subunit R (EC 3.1.21.3)	Pseudomonas stutzeri DSM 4166	YP_005937550.1	0	927	1056
**PSF113_5187**	CDS	-	6009929..6010915	328		FIG00958850: hypothetical protein	Pseudomonas fluorescens BBc6R8	ZP_15603748.1	0	279	321
**PSF113_5191**	CDS	-	6013571..6016819	1082		COG0553: Superfamily II DNA/RNA helicases, SNF2 family	Pseudomonas extremaustralis 14-3 substr. 14-3b	ZP_10435057.1	0	1011	1082
**PSF113_5192**	CDS	-	6016819..6018447	542		putative; ORF located using Glimmer/Genemark	Pectobacterium carotovorum subsp. carotovorum PCC21	YP_006647908.1	5E-152	229	529
**PSF113_5193**	CDS	-	6018444..6019187	247		COG2885: Outer membrane protein and related peptidoglycan-associated (lipo)proteins	Pseudomonas extremaustralis 14-3 substr. 14-3b	ZP_10435055.1	5E-177	238	247
**PSF113_5194**	CDS	-	6019201..6021027	608		putative membrane protein	Hafnia alvei ATCC 51873	ZP_09376790.1	0	379	721
**PSF113_5316**	CDS	+	6149144..6149848	234		hypothetical protein	Pseudomonas aeruginosa PAO1	NP_254115.1	17	40	125
**PSF113_5379**	CDS	-	6220451..6220741	96		Cro-like protein	Pseudomonas fluorescens	ACT32384.1	5E-63	96	96
**PSF113_5380**	CDS	-	6220758..6220916	52		hypothetical protein	Pseudomonas fluorescens	ACT32385.1	3E-29	51	52
**PSF113_5636**	CDS	-	6504147..6505682	511		multidrug resistance efflux protein	Bacillus cereus BAG1X1-2	ZP_17364789.1	3E-163	261	500
**PSF113_5637**	CDS	-	6505682..6505957	91		hypothetical protein	Photorhabdus asymbiotica	YP_003040022.1	3E-10	29	84
**PSF113_5638**	CDS	-	6506559..6508595	678		4-amino-4-deoxychorismate synthase, amidotransferase component , aminase component	Rhodococcus erythropolis SK121	ZP_04383376.1	7E-176	302	673
**PSF113_5640**	CDS	-	6510201..6510953	250	prmC	PrmC	Bacillus cereus BAG1X1-2	ZP_17364784.1	2E-75	110	235
**PSF113_5641**	CDS	-	6511046..6512476	476	forZ	N-formimidoyl fortimicin A synthase	Pseudomonas fluorescens SBW25	YP_002871412.1	0	293	431
**PSF113_5672**	CDS	-	6543144..6543617	157		TonB-like protein	Pseudomonas sp. GM78	ZP_10618829.1	6E-46	80	158
**PSF113_5730**	CDS	+	6602672..6603031	119		Mobile element protein	Pseudomonas syringae pv. tomato str. DC3000	NP_793006.1	3E-79	117	119
**PSF113_5813**	CDS	+	6705413..6705991	192	tnpR	TnpR	Pseudomonas putida	CAB54047.1	4E-84	118	146
**PSF113_5903**	CDS	-	6824047..6825018	323	tctC2	Tricarboxylate transport protein TctC	Pseudomonas sp. S9	ZP_09711490.1	0	246	327
