# Supplementary material for: Genome sequence reveals that Pseudomonas fluorescens F113 possesses a large and diverse array of systems for rhizosphere function and host interaction
Source: BMC Genomics. 2013 Jan 25;14:54. doi: 10.1186/1471-2164-14-54 (PMC3570484; doi:10.1186/1471-2164-14-54)
Supplement: Additional file 4 — Distribution of denitrification pathway in strains belonging to the P. fluorescens group. [file 1471-2164-14-54-S4.pdf]

SubGroup	Organism	nar	nir	nor1	nor2	nos1	nos2
	P. fluorescens F113	■	■	■	■	■	■
	P. fluorescens Wood1R	■	■	■		■	■
I	*P. brassicacearum* NFM421	■	■	■	■	■	■
	P. brassicacearum Q8r1-96	■	■	■		■	■
	P. fluorescens Q2-87	■	■	■		■	■
	P. fluorescens Pf0-1						
	Pseudomonas sp GM25						
	P. fluorescens R124						
	Pseudomonas sp GM30						
II	*Pseudomonas sp* GM80						
	P. fluorescens NZ011						
	Pseudomonas sp GM16						
	Pseudomonas sp GM24						
	Pseudomonas sp R62						
	Pseudomonas sp GM49						
	Pseudomonas sp GM48						
	Pseudomonas sp GM74						
	Pseudomonas sp GM33						
	Pseudomonas sp GM55						
	Pseudomonas sp GM78						
	Pseudomonas sp GM21						
III	*Pseudomonas sp* GM50						
	Pseudomonas sp GM102						
	Pseudomonas sp GM79						
	Pseudomonas sp GM18						
	Pseudomonas sp GM41	■	■	■		■	■
	P. mandelii JR-1	■	■	■		■	■
	P. fluorescens NCIMB 11764						
	Pseudomonas sp GM67	■	■	■		■	■
	Pseudomonas sp GM60	■	■	■		■	■
	P. protegens Pf-5						
	P. fluorescens Wayne1						
	P. fluorescens NZ17						
IV	*P. chlororaphis* 30-84						
	Pseudomonas sp GM17	■		■			
	P. chlororaphis O6	■		■			
	P. chlororaphis GP72	■		■			
	P. tolaasii NCPPB 2192						
	P. tolaasii PMS117						
	Pseudomonas sp Ag1						
	Pseudomonas sp PAMC 25886						
	P. extremaustralis 14-3 substr 14-3b	■		■		■	■
	P. fluorescens A506						
V	*P. fluorescens* SS101						
	Pseudomonas sp BG33R						
	P. fluorescens NZ007						
	P. fluorescens WH6	■	■	■			
	P. fluorescens SBW25						
	P. fluorescens NZ052						
	Pseudomonas sp R81	■					
