# Supplementary material for: Genome sequence reveals that Pseudomonas fluorescens F113 possesses a large and diverse array of systems for rhizosphere function and host interaction
Source: BMC Genomics. 2013 Jan 25;14:54. doi: 10.1186/1471-2164-14-54 (PMC3570484; doi:10.1186/1471-2164-14-54)
Supplement: Additional file 5 — Distribution of chemotaxis systems in strains belonging to theP. fluorescensgroup. [file 1471-2164-14-54-S5.pdf]

SubGroup	Organism	Wsp	Chp	Che1	Che2	Che3
I	*P. fluorescens* F113	■	■	■	■	■
	P. fluorescens Wood1R	■	■	■	■	■
	P. brassicacearum NFM421	■	■	■	■	■
	P. brassicacearum Q8r1-96	■	■	■	■	
	P. fluorescens Q2-87	■	■	■	■	
II	*P. fluorescens* Pf0-1	■	■	■		
	Pseudomonas sp GM25	■	■	■		
	P. fluorescens R124	■	■	■		
	Pseudomonas sp GM30	■	■	■		
	Pseudomonas sp GM80	■	■	■		
	P. fluorescens NZ011	■	■	■		
	Pseudomonas sp GM16	■	■	■		
	Pseudomonas sp GM24	■	■	■		
	Pseudomonas sp R62	■	■	■		
III	*Pseudomonas sp* GM49	■	■	■		
	Pseudomonas sp GM48	■	■	■		
	Pseudomonas sp GM74	■	■	■		
	Pseudomonas sp GM33	■	■	■		
	Pseudomonas sp GM55	■	■	■		
	Pseudomonas sp GM78	■	■	■		
	Pseudomonas sp GM21	■	■	■		
	Pseudomonas sp GM50	■	■	■		
	Pseudomonas sp GM102	■	■	■		
	Pseudomonas sp GM79	■	■	■		
	Pseudomonas sp GM18	■	■	■		
	Pseudomonas sp GM41	■	■	■		
	P. mandelii JR-1	■	■	■		
	P. fluorescens NCIMB 11764	■	■	■		
	Pseudomonas sp GM67	■	■	■		
	Pseudomonas sp GM60	■	■	■		
IV	*P. protegens* Pf-5	■	■	■		
	P. fluorescens Wayne1	■	■	■		
	P. fluorescens NZ17	■	■	■		
	P. chlororaphis 30-84	■	■	■		
	Pseudomonas sp GM17	■	■	■		
	P. chlororaphis O6	■	■	■		
	P. chlororaphis GP72	■	■	■		
V	*P. tolaasii* NCPPB 2192	■	■	■		
	P. tolaasii PMS117	■	■	■		
	Pseudomonas sp Ag1	■	■	■		
	Pseudomonas sp PAMC 25886	■	■	■		
	P. extremaustralis 14-3 substr 14-3b	■	■	■	■	■
	P. fluorescens A506	■	■	■		
	P. fluorescens SS101	■	■	■		
	Pseudomonas synxantha BG33R	■	■	■		
	P. fluorescens NZ007	■	■	■		
	P. fluorescens WH6	■	■	■	■	
	P. fluorescens SBW25	■	■	■	■	
	P. fluorescens NZ052	■	■	■		
	Pseudomonas sp R81	■	■	■		
